# Resilient Calvarial Bone Marrow Supports Retinal Repair in Type 2 Diabetes

**DOI:** 10.1002/advs.202519680

**Published:** 2026-01-04

**Authors:** Bright Asare‐Bediako, Sergio Li Calzi, Julia G. Behnsen, Ram Prasad, Magdalena Blaszkiewicz, Yvonne Adu‐Rutledge, Robert F. Rosencrans, Jason L. Floyd, Ashley Rennhack, Denise Stanford, Anna Lin, Todd A. Lydic, Carl M. Sheridan, Michael E. Boulton, Kristy L. Townsend, Julia V. Busik, Maria B. Grant

**Affiliations:** ^1^ Department of Ophthalmology and Visual Sciences School of Medicine The University of Alabama at Birmingham Birmingham Alabama USA; ^2^ Department of Ophthalmology School of Medicine Stanford University Palo Alto California USA; ^3^ Department of Materials Design and Manufacturing Engineering School of Engineering University of Liverpool Liverpool UK; ^4^ Department of Neurological Surgery The Ohio State University Columbus Ohio USA; ^5^ Schepens Eye Research Institute Department of Ophthalmology Harvard Medical School Boston Massachusetts USA; ^6^ Department of Medicine Cystic Fibrosis Research Center The University of Alabama at Birmingham Birmingham USA; ^7^ Collaborative Mass Spectrometry Core Michigan State University East Lansing Michigan USA; ^8^ Department of Eye and Vision Science Institute of Life Course and Medical Sciences University of Liverpool Liverpool UK; ^9^ Department of Biochemistry and Physiology University of Oklahoma Oklahoma City Oklahoma USA

**Keywords:** calvarium marrow, ischemia‐reperfusion, long bones, myeloid angiogenic cells, neutrophils, retina, skull, stem/progenitor cells

## Abstract

Using micro‐computed tomography, we identified a network of skull channels in the calvarium of type 2 diabetic (T2D) mice that remained structurally intact and numerically stable despite long‐standing disease. The retention of calvaria bone marrow structural integrity was associated with preserved hematopoietic capacity under chronic diabetic conditions, which was not observed in the bone marrow of long bones. A distinctive feature of the calvarial bone marrow compartment was its direct exposure to cerebrospinal fluid (CSF), a property not shared by tibial bone marrow. To characterize the biochemical environment of the murine calvarium, we profiled oxysterols in CSF using mass spectrometry. The CSF exhibited elevated levels of neurotrophic and anti‐inflammatory oxysterols, including 22‐hydroxycholesterol (22‐OHC) and 27‐hydroxycholesterol (27‐OHC). To assess whether this protective oxysterol signature was conserved in humans, we analyzed CSF samples from diabetic and non‐diabetic individuals with obesity‐associated idiopathic intracranial hypertension (IIH). Human CSF contained 7α‐hydroxy‐3‐oxo‐4‐cholestenoic acid (7‐HOCA), a metabolite of 27‐OHC, supporting the conservation of this neuroprotective profile across species. Given the anatomical proximity of the calvarium to the eye, we hypothesized that calvaria bone marrow may serve as a reservoir for immune cells recruited to the injured or infected retina. The calvaria bone marrow was the predominant source of myeloid angiogenic cells (MACs) and neutrophils, mobilizing these cells at levels approximately 20‐fold higher than long bones. These findings demonstrate that calvarial bone marrow plays a critical role in retinal immune defense, while maintaining both structural integrity and functional capacity despite chronic T2D.

## Graphical abstract

Skull bone (calvarium) marrow in diabetic mice stay structurally intact and keeps making blood cells, unlike the bone marrow of the leg bones. The skull marrow is exposed to cerebrospinal fluid (CSF), which contains protective molecules called oxysterols. Similar molecules are found in human CSF, suggesting this protective environment exists across species. The skull marrow also sends far more immune cells to the retina (about 20 times more than leg bones), helping defend against injury or infection in the eye. In short, skull bone marrow remains strong in diabetes and plays a key role in eye health.

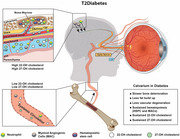

**Abbreviations**
22‐OHC: 22‐hydroxycholesterol
27‐OHC: 27‐hydroxycholesterol
AQP4: Aquaporin 4
BM: Bone marrow
CHIP: Clonal haematopoiesis of intermediate potential
CNS: Central nervous system
CSF: Cerebrospinal fluid
CACs: Circulating angiogenic cells.
DMHCA: N, N‐dimethyl‐3β‐hydroxy‐cholenamide
HSCs: Haematopoietic stem cells
HSPCs: Haematopoietic stem and progenitor cells
I/R: Ischemia reperfusion injury
KikGR: Kikume Green‐Red photoconvertible fluorescent protein
IIH: Idiopathic Intracranial Hypertension
LXR: Liver X receptor
LT‐HSCs: Long‐term haematopoietic stem cells
MACs: Myeloid angiogenic cells
Micro‐CT: Micro computed tomography
ST‐HSCs: Short‐term haematopoietic stem cells
T2D: Type 2 diabetes

## 1 Introduction

The discovery of bone channels connecting cerebrospinal fluid (CSF) to the skull and vertebral bone marrow (BM) compartments has revealed a novel route for leukocyte trafficking, enabling immune surveillance of the central nervous system (CNS) [1]. Unlike distal BM niches, the calvarial BM can mirror brain pathology, as demonstrated by the presence of tumor‐reactive CD8⁺ effector cells in glioblastoma [2]. These osseous conduits, commonly referred to as “skull channels”, contain both arterioles and perivascular spaces that facilitate the exchange of cells, nutrients, oxygen, and solutes between the CSF, systemic circulation, and calvaria BM. The directional migration of hematopoietic cells, particularly neutrophils, from the calvarium into the brain in response to injury such as stroke underscores the functional connectivity of this compartment [1].

Cai and others corroborated these findings using Lyz2‐EGFP mice and skull transplantation experiments, demonstrating the migration of skull‐derived myeloid cells into the underlying meninges [3, 4]. Complementary work by Herisson et al. employed real‐time imaging of organ bath preparations to visualize monocyte and neutrophil trafficking through CD31⁺ vessels within skull channels toward the meninges [1]. Collectively, these studies support the existence of a neuroimmune signaling axis, wherein CSF directly accesses the calvarial BM via skull channels, delivering context‐dependent cues that modulate hematopoietic activity within this niche [5].

Other unique regions within the CNS also feature close proximity between CSF and adjacent tissues, allowing CSF to influence local function, most notably the neural retina and optic nerve. CSF can enter and exit these ocular structures via perivascular spaces surrounding the central retinal vessels, forming part of an aquaporin‐4 (AQP4)‐dependent ocular glymphatic system [6]. This system enables CSF to surround the optic nerve, deliver nutrients, and facilitate immune cell trafficking to the retina [7]. The presence of CSF near the optic nerve and within calvaria bone marrow suggests that CSF components may directly influence local tissues, including the retina, brain, and hematopoietic cells of the calvarium [5]. These insights raise fundamental questions about how acute injury or chronic disease may disrupt the homeostatic functions of CSF. In particular, it remains unclear whether metabolic conditions such as diabetes alter the CSF and hematopoietic output of the calvarial BM, potentially impacting neuroimmune responses.

Diabetes [8], aging [9], and obesity [10] negatively impact hematopoiesis by promoting the accumulation of fatty bone marrow [10], which contributes to clonal hematopoiesis of intermediate potential (CHIP), a condition that has gained significant attention in recent years. Diabetes further depletes hematopoietic stem and progenitor cells (HSPCs), skews hematopoiesis toward myeloid‐biased lineages, and impairs the mobilization of reparative cells following injury [11]. However, most animal and human studies rely on HSPCs isolated from long bones rather than the calvarium, primarily due to the ease of isolation from long bones and the technical challenges associated with harvesting cells from the calvarium and vertebrae.

Both acute and chronic tissue injury trigger the release of cytokines and regulatory factors into the circulation, initiating the mobilization of reparative hematopoietic cells [12]. Neutrophils are typically the first responders to injury and infection [1], while myeloid angiogenic cells (MACs) represent another reparative population that contributes to vascular restoration, primarily through the secretion of pro‐angiogenic growth factors [13, 14]. Aging and diabetes impair this reparative process by disrupting MAC mobilization from the bone marrow [15] and diminishing their functional capacity [16], resulting in inadequate tissue repair.

Oxysterols are oxygenated derivatives of cholesterol that play essential roles in regulating lipid metabolism, immune function, and cellular signaling [17]. They contribute to cholesterol homeostasis by acting as feedback inhibitors of cholesterol synthesis and by activating liver X receptors (LXRs), nuclear receptors that control the expression of genes involved in cholesterol transport and efflux. Beyond their metabolic functions, oxysterols such as 22‐hydroxycholesterol (22‐OHC) and 27‐hydroxycholesterol (27‐OHC) exhibit potent immunomodulatory properties, influencing immune cell migration, macrophage polarization, and dendritic cell activity [18]. In the central nervous system (CNS), oxysterols can cross the blood‐brain barrier and participate in neuroprotecton by reducing neuroinflammatory processes. For example, 24S‐hydroxycholesterol facilitates cholesterol clearance from neurons, contributing to CNS lipid balance [19]. Dysregulation of oxysterol levels has been implicated in a variety of diseases, including atherosclerosis, neurodegenerative disorders, metabolic syndromes, and cancer [20]. Importantly, oxysterol production is often tissue‐specific and context‐dependent, allowing these molecules to act as localized signaling agents. In the calvaria BM, we hypothesize that oxysterols may influence immune responses in adjacent tissues such as the retina, highlighting a potential neuroimmune interface mediated by CSF‐derived lipid signals.

In this study, we demonstrate that the delicate architecture of skull channels, which enable CSF access to the calvaria BM, remains preserved in the context of chronic metabolic disease, highlighting their remarkable structural resilience. Both diabetic and control mice exhibited similarly elevated levels of neurotrophic and anti‐inflammatory oxysterols, including 22‐OHC and 27‐OHC, in the CSF compared to blood. These findings support the notion that CSF‐derived oxysterols are selectively accessible to the calvaria BM, but not to the long bone compartment. Using Kikume Green‐Red (KikGR) reporter mice, we performed photoconversion of either the calvarial or long bone BM compartments and observed that, following retinal injury, the calvarium mobilized more neutrophils and myeloid angiogenic cells (MACs) to the injured retina than the long bones. Collectively, these data provide compelling evidence that the calvaria BM retains its structural integrity and hematopoietic capacity under chronic diabetic conditions, in contrast to long bones. Importantly, skull channel architecture, CSF composition, and robust hematopoiesis are maintained in diabetes, positioning the calvarium as a critical neuroimmune interface that not only protects the brain but also contributes to retinal immune defense.

## 2 Results

### 2.1 Structural Features of the Skull Channels in Wild Type and Diabetic Mice

**FIGURE 1:**
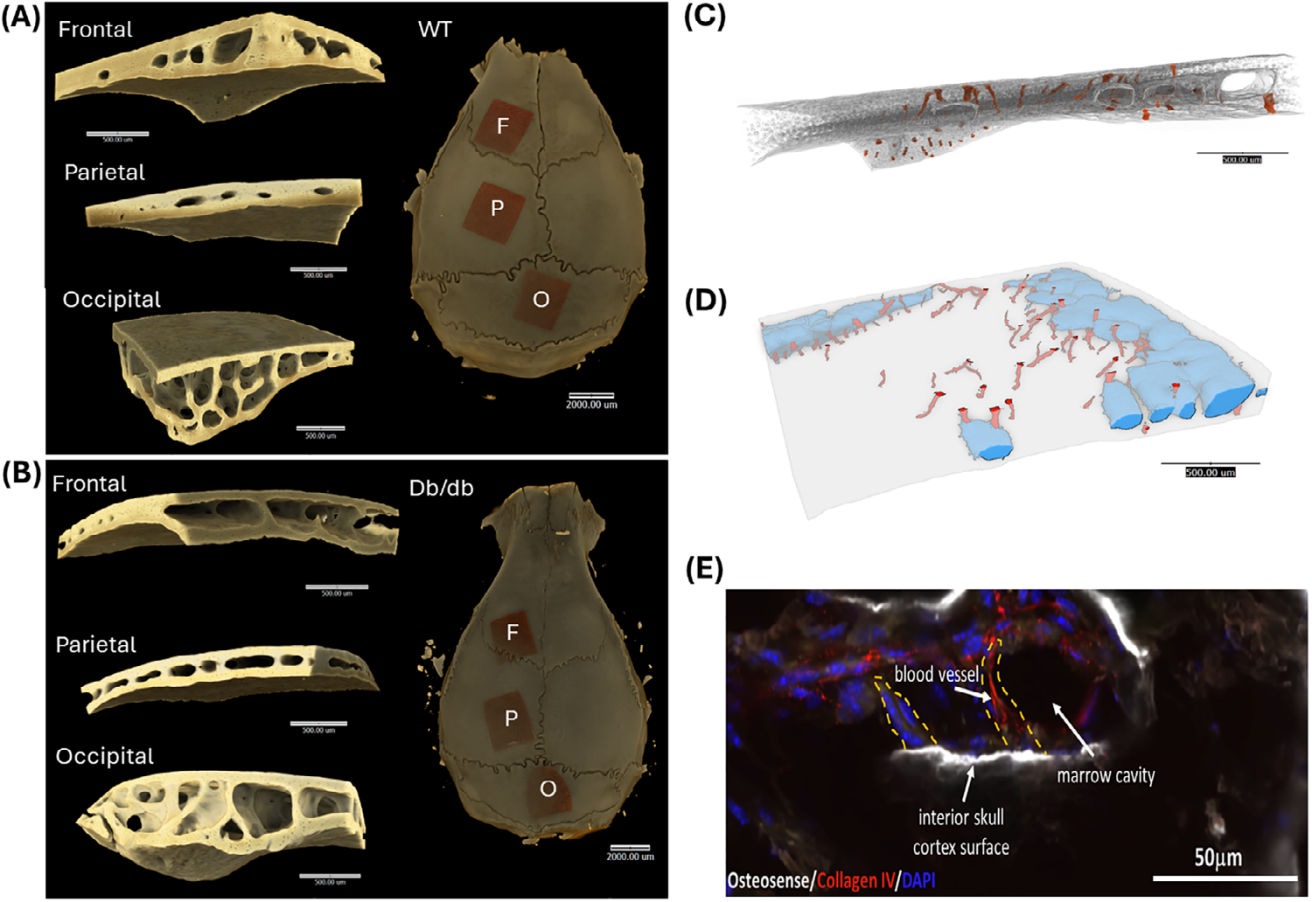
Ex vivo micro‐CT and immunofluorescence imaging of mouse calvarium. (A, B) Micro‐CT volume renderings of the calvarium from (A) WT and (B) db/db mice, highlighting three regions of interest: frontal, parietal, and occipital. (C) High‐resolution rendering of the frontal region (voxel size: 2 µm), with skull channels outlined in red. (D) Surface mesh of the same region showing bone marrow cavities (blue) and skull channels (red). (E) Immunofluorescence microscopy of skull channels stained with collagen IV, highlighted by yellow dashed lines. n = 3 mice per group.

Using micro‐computed tomography (micro‐CT), we generated detailed renderings of the frontal skull region in wild‐type (WT) (Figure 1A) and diabetic (db/db) mice (Figure 1B), revealing channels that penetrate the calvaria BM cavity (Figures 1C,D; S1A,B; Video S1). These skull channels remain structurally intact in diabetic mice, underscoring their resilience in chronic metabolic disease. Immunofluorescence staining confirmed the presence of these channels using OsteoSense, a near‐infrared fluorescent agent that labels bone calcification. Blood vessels within the channels were visualized using collagen‐IV antibody staining (Figure 1E).

Additional renderings of the occipital skull bone (Figure S2) revealed channels extending from the inner skull cavity into the BM compartment, facilitating CSF entry into the calvarium. Bone channels were observed to connect BM compartments directly (green, Figure S2A), while dorsal channels (purple) emerged from the large BM cavity and tracked upward (Figure S2B; Video S2). Similarly, the parietal skull region displayed prominent ventral (light purple) and dorsal channels (orange) (Figure S2C; Video S3). Together, these findings confirm the existence of a complex, interconnected network of bone channels that enable communication between the CSF and BM compartments across multiple regions of the calvarium in both WT and diabetic mice. We compared skull channel length and width in WT and db/db mice at 4 and 8 months of age, corresponding to 2 and 6 months of diabetes progression in db/db mice. Measurements focused on the frontal, parietal, and occipital regions of the calvarium. In WT mice, channel length increased with age across all regions (Figure S3A–C). In contrast, 8‐month‐old db/db mice exhibited a significant reduction in frontal channel length only (Figure S3A).

We next reasoned that combining data from different calvaria regions (Figure S3D) would provide an overall assessment of calvaria bone characteristics rather than region‐specific differences. While regional variability exists, our primary objective was to capture the global effect within the calvarium, which is relevant to the systemic nature of diabetes. Thus, when data from all regions were combined (Figure S3D), both WT and db/db mice showed an overall increase in channel length at 8 months compared to 4 months, suggesting that age‐related elongation of skull channels occurs regardless of diabetic status, with region‐specific vulnerability in the frontal calvarium of diabetic mice. This selective reduction in frontal channel length in 8‐month db/db mice may reflect region‐specific susceptibility of calvaria bone, as the frontal region is highly vascularized and metabolically active, which could make it more sensitive to diabetes‐induced changes in bone remodeling and marrow niche function. Additionally, localized differences in mechanical loading and osteoblast/osteoclast activity across calvaria regions may contribute to this pattern.

In contrast, channel width remained stable in the frontal and parietal regions across all groups (Figure S3E,F). In the occipital region, 8‐month WT mice showed increased width, while db/db mice at both ages resembled 4‐month WT mice (Figure S3G). Frontal channel widths ranged from 6 to 26 µm in 4‐month mice and 10 to 24 µm in 8‐month WT mice, with db/db mice showing similar distributions (Figure S3H). Some openings appeared as surface indentations without underlying cavities. Among the regions examined, the occipital calvarium consistently exhibited the longest skull channels, measuring approximately 300–400 µm, likely reflecting its distinct anatomical structure. Overall, 8‐month‐old db/db mice did not show major structural alterations in calvaria channels compared to WT controls, indicating that skull channel architecture remains largely preserved despite chronic metabolic disease.

**FIGURE 2:**
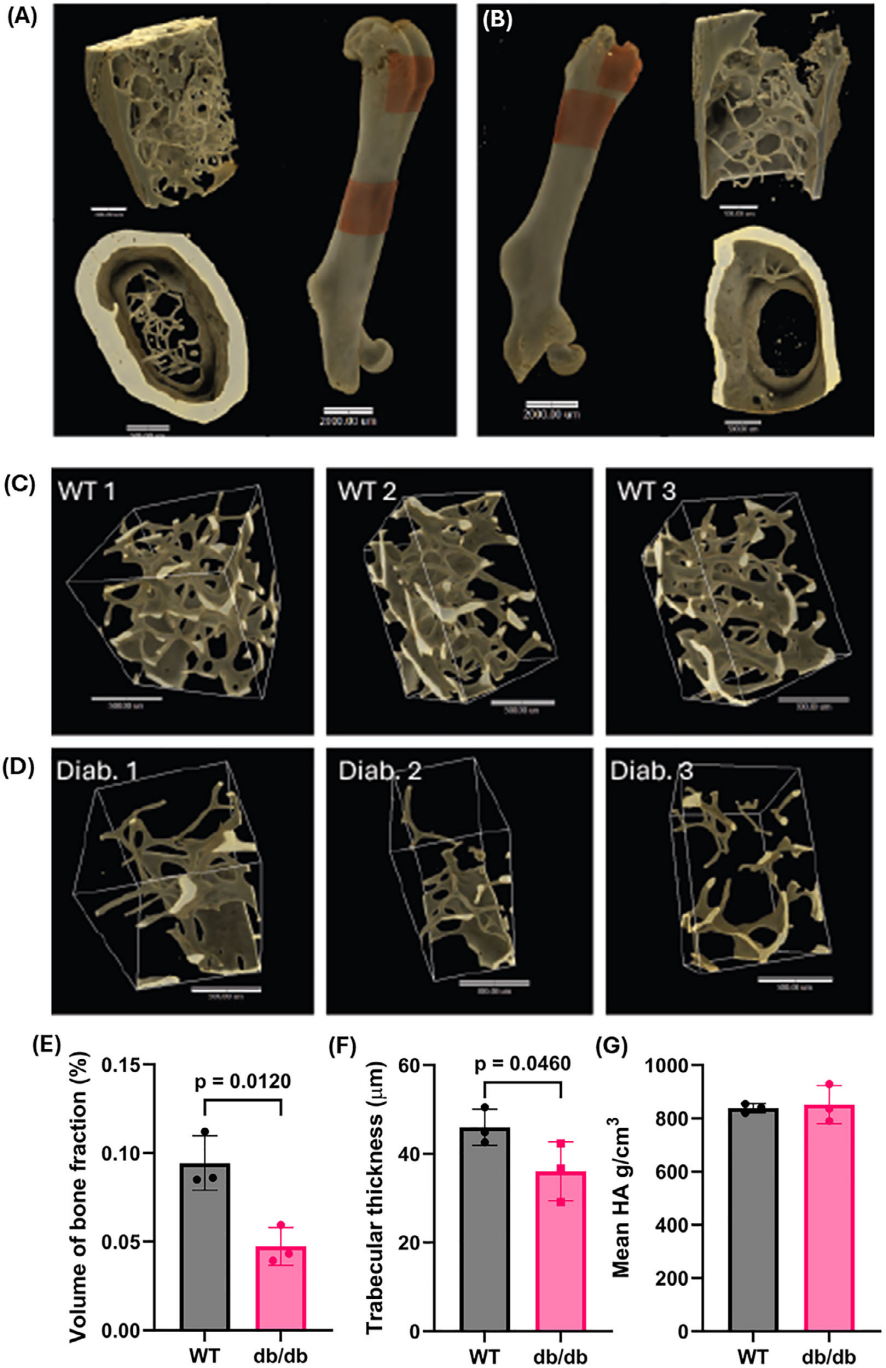
Micro‐CT analysis of femoral bone structure in WT and db/db mice. (A, B) Regions of interest in the metaphysis of femurs from WT and age‐matched db/db mice selected for micro‐CT imaging. (C) Cross‐sectional images of trabecular bone in WT mice (n = 3). (D) Corresponding cross‐sections in db/db mice (n = 3) showing markedly reduced trabecular bone. (E–G) Quantitative analysis of bone parameters: bone volume fraction (E), trabecular thickness (F), and hyaluronic acid content (G) in WT and diabetic mice. n = 3 mice per group.

Micro‐CT analysis of femurs revealed a marked reduction in trabecular number in db/db mice compared to WT controls (Figure 2A–D). Diabetic mice also exhibited a significant decrease in bone volume fraction relative to WT mice (Figure 2E). Trabecular thickness showed a decline in db/db mice (Figure 2F). No changes were observed in hyaluronic acid (HA) content within the bone matrix between groups (Figure 2G), suggesting that while structural deterioration occurs in diabetic long bones, extracellular matrix composition remains largely unaffected. Since HA content is preserved, this may further suggest that non‐mineralized components of bone, such as the extracellular matrix and marrow niche, are less affected compared to the mineralized matrix. HA is primarily located in the organic phase of bone and contributes to hydration, cell signaling, and matrix organization rather than hydroxyapatite mineralization [21, 22]. Therefore, preserved HA may indicate selective vulnerability of the mineralized matrix in long bones, which relies more heavily on osteoblast activity and calcium‐phosphate homeostasis.

### 2.2 Hematopoietic Cell Function in Calvarium vs. Long Bones by Competitive Repopulation Assay

To begin to understand if there were functional differences in hematopoietic cells obtained from the calvarium compared to the long bones, we performed the competitive repopulation assay [23, 24]. The competitive repopulation assay is a powerful method to evaluate the hematopoietic potential of donor cells by assessing both short‐term and long‐term reconstitution of the host's BM. This approach involves co‐transplantation of donor and competitor cells into a conditioned recipient, allowing direct comparison of their ability to repopulate hematopoietic compartments over time. We used CD45.1 donor cells and transplanted them into lethally irradiated CD45.2 recipient mice. The differential expression of CD45 allelic markers enables precise tracking of donor‐versus host‐derived hematopoietic cells by flow cytometry. This design provides a framework for quantifying engraftment efficiency, lineage contribution, and durability of hematopoietic stem and progenitor cell function.

At P7, P14, and P21, HSPCs generated from either the calvarium BM or tibia BM were used to transplant lethally irradiated host mice. Biweekly, the host blood was assessed for repopulation potential by donor cells using flow cytometry to measure CD45.1 cells (Figure S4A). When donor cells were obtained from P7 pups from either compartment [calvarium BM (gray line) or tibial‐BM (pink line)], the number of CD45.1 cells in the peripheral blood was the same by 6 weeks (Figure S4B). However, prior to this time point, the calvarium BM‐derived donor cells were slower to fully repopulate the CD45.2 mice with mature cells (CD45.1 cells) than the tibial ‐BM‐derived donor cells. This identical pattern was observed when the transplanted HSPC were obtained from P14 pups (Figure S4C) or P21 pups (Figure S4D), indicating that during early development, the HSPC from tibial BM repopulate a lethally irradiated host faster than HSPCs from the calvarium ‐BM.

Next, we evaluated the host mice at 16 weeks post transplantation (PT). This is the time point considered as stable reconstitution (Figure S5). At the time of stable reconstitution, significantly higher percentages of long‐term HSCs (LT‐HSCs) (Figure S5A), short‐term HSCs (ST‐HSCs) (Figure S5B), and multipotent progenitors (MPPs) (Figure S5C) were present in the calvarium BM compared to the tibial BM of mice for all donor cohorts (P7, P14, P21). Collectively, these findings support that during early postnatal development, the calvarium matures more slowly than the long bones. However, once maturity is achieved, the calvarium generates higher percentages of HSPC.

### 2.3 Haematopoiesis in the Calvarium BM Compartment is Resilient to the Impact of Chronic Diabetes

**FIGURE 3:**
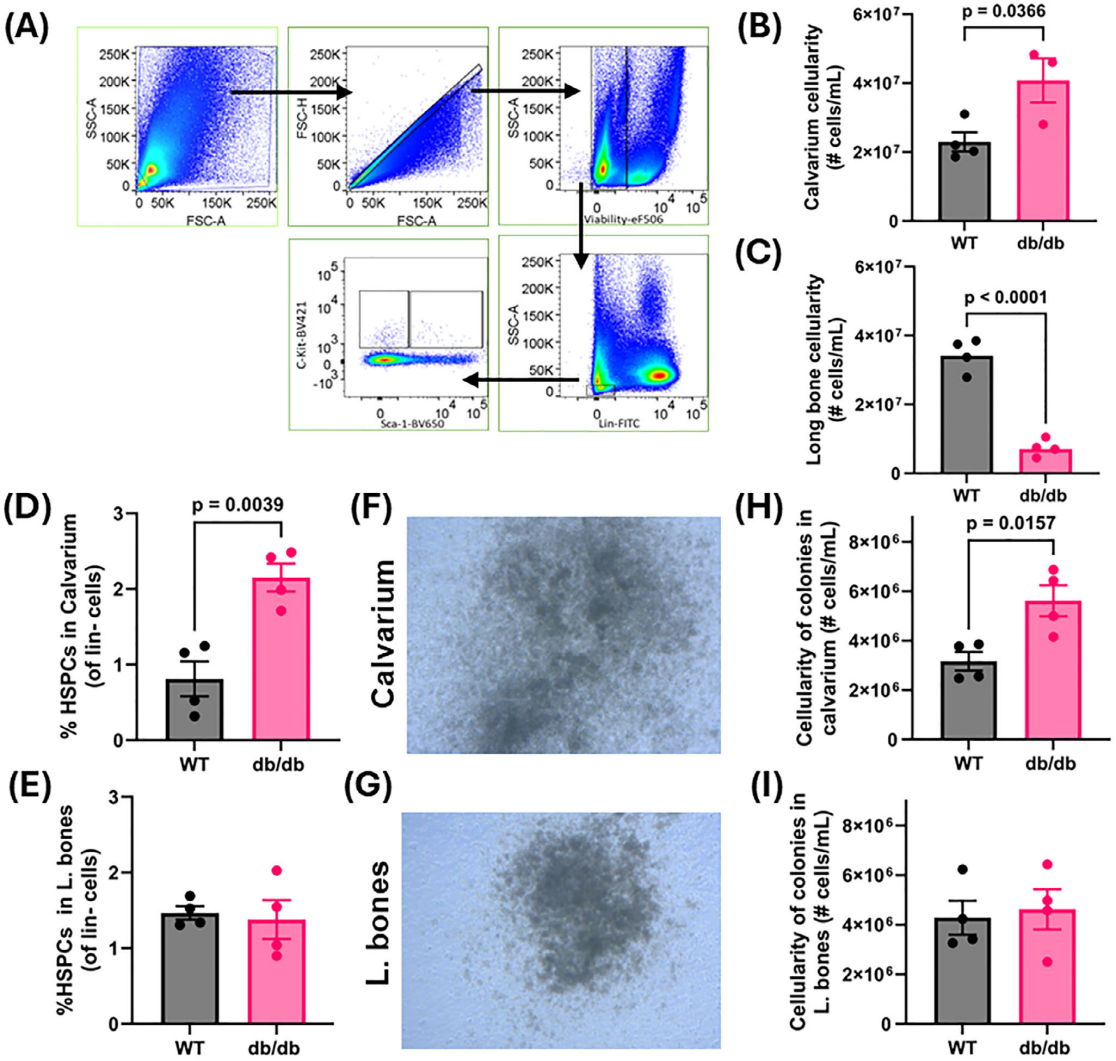
Flow cytometric analysis of hematopoietic stem/progenitor cells (HSPCs) in the calvarial and long bone marrow of *db/db* and control mice. (A) Representative flow cytometry plots illustrating the gating strategy used to identify Lin^−^/Sca‐1^−^/c‐Kit⁺ (LS‐K) and Lin^−^/Sca‐1⁺/c‐Kit⁺ (LSK) populations. LS‐K and LSK cells were combined and analyzed as HSPCs. (B, C) Bar graphs quantifying total cellularity in the BM of calvaria and long bones, respectively. (D, E) Bar graphs showing the relative proportions of HSPCs in calvarium vs. long bone marrow. (F, G) Representative images of colony‐forming units derived from calvarium and long bone marrow cells. (H, I) Quantification of colony cellularity. At 12 days post‐seeding, single‐cell suspensions from harvested colonies revealed a significant increase in cellularity in colonies derived from db/db calvaria compared to controls, whereas no significant difference was observed in colonies from long bones. n = 3–7 mice per group.

To characterize hematopoietic changes in diabetic conditions, we next performed flow cytometric analysis to quantify HSPCs within the BM (Figure 3A) and measure total cellularity across distinct BM compartments (Figure 3B,C). Notably, BM harvested from the calvarium of db/db mice exhibited significantly increased cellularity compared to age‐matched WT controls (Figure 3B), suggesting region‐specific alterations in marrow composition under diabetic stress.

In contrast, the long bone compartment of db/db mice showed a marked reduction in cellularity relative to WT mice (Figure 3C). The percentage of HSPCs was significantly elevated in the calvaria BM of diabetic mice compared to WT (Figure 3D), whereas HSPC percentages in the long bones were comparable between the two groups (Figure 3E). To further investigate these findings, we conducted ex vivo colony‐forming assays (Figure 3F–I). Colonies were expanded, harvested, and quantified from calvaria and long bone BM of both WT and diabetic cohorts. The calvarium of db/db mice produced larger colonies with higher cellularity than those from WT calvarium (Figure 3F,H), while colonies derived from long bones showed similar cellularity between WT and diabetic mice (Figure 3G,I). Collectively, these results strongly support that the calvaria BM compartment is resistant to the adverse effects of chronic diabetes and may even compensate for the changes in the long bones. The calvarium BM maintains enhanced cellularity, increased colony‐forming capacity in vitro, and a greater abundance of HSPCs compared to the diabetic long bone compartment.

**FIGURE 4:**
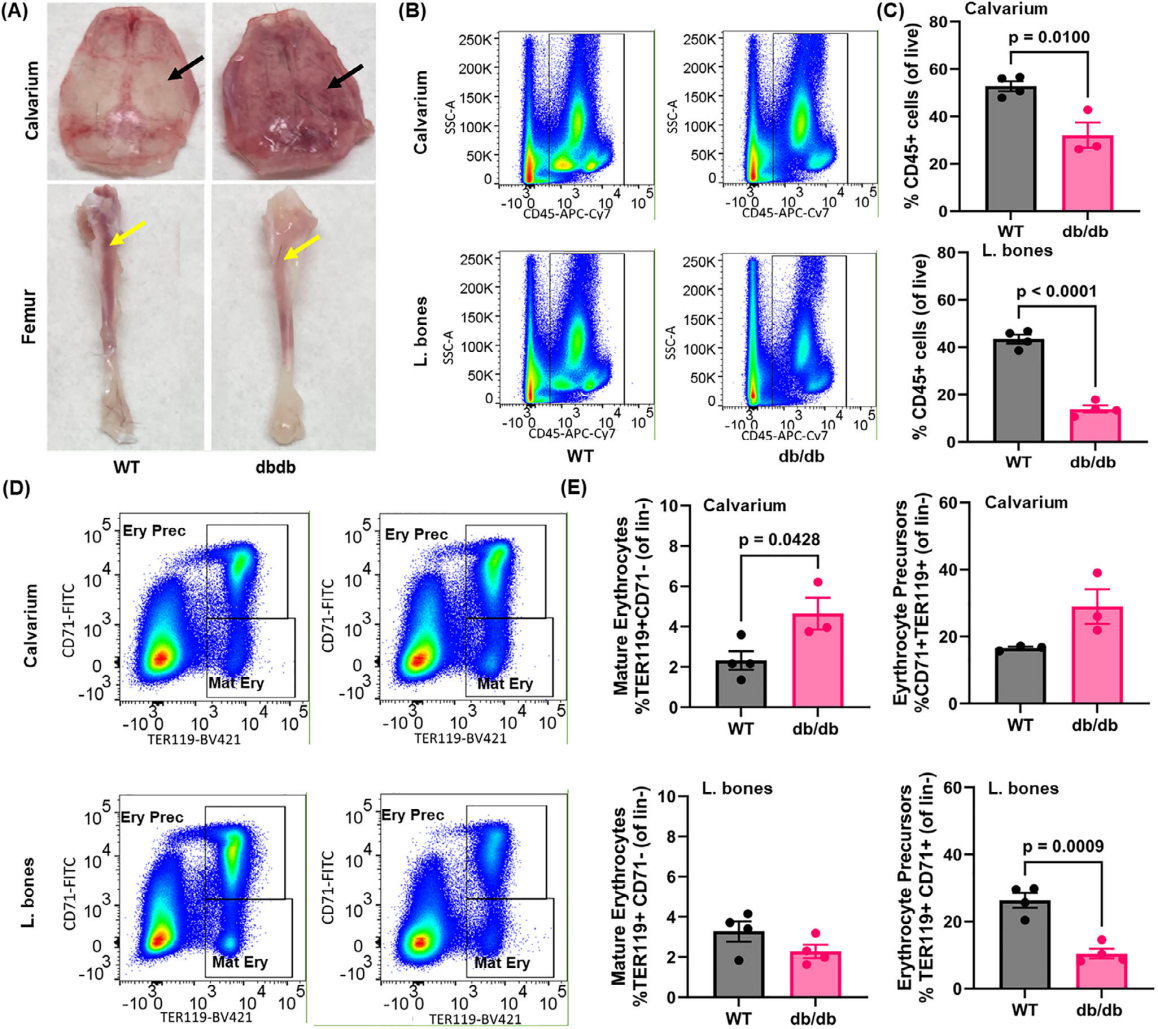
Calvarium and long bones demonstrate unique site specific differences in BM composition. (A) Representative images of calvaria and tibia from WT and db/db mice. In db/db mice, the calvarium displays increased red marrow and reduced fat content (black arrows), whereas the femur shows decreased red marrow and increased fat accumulation compared to WT controls (yellow arrows). (B–E) Flow cytometric analysis of hematopoietic and erythroid populations in calvarium and long bone marrow. (B) Representative flow plots and (C) quantification of CD45⁺ cells from calvaria and long bones of WT and db/db mice. (D) Representative flow plots and (E) quantification of erythroid lineage cells, including erythroid precursors (Ery Prec; CD71⁺TER119⁺) and mature erythrocytes (Mat Ery; CD71^−^TER119⁺), from calvaria and long bones of WT and db/db mice. n = 3–7 mice per group.

Interestingly, the calvarium of 12‐month‐old db/db mice appeared visibly redder than that of age‐matched WT mice (Figure 4A, top right vs. top left), suggesting enhanced erythropoietic activity. In contrast, the long bone marrow of db/db mice exhibited a yellow appearance in the epiphysis and metaphysis (Figure 4A, bottom right), indicative of adipocyte‐rich marrow, compared to the more hematopoietically active long bone marrow of WT mice (Figure 4A, bottom left).

Flow cytometric analysis of mature hematopoietic populations revealed a decreased frequency of CD45⁺ cells, representing total hematopoietic cells, in both the calvaria and long bone marrow compartments of diabetic mice compared to controls (Figure 4B,C).

However, the degree of reduction was significantly less in the calvarium (Figure 4C, top) than in the long bones (Figure 4C, bottom), consistent with the preserved hematopoietic potential. To further investigate erythropoiesis, we quantified erythroid lineage cells in each compartment (Figure 4D,E). The calvarium of db/db mice contained significantly more mature erythrocytes (TER119⁺CD71^−^) compared to WT (Figure 4E, top left), while changes in the erythroid precursor levels (TER119⁺CD71⁺) did not achieve significance (Figure 4E, top right). In contrast, the long bones of db/db mice showed a non‐significant reduction in mature erythrocytes (Figure 4E, bottom left) but a significant decrease in erythroid precursors (Figure 4E, bottom right). These findings suggest that, despite systemic metabolic stress, the calvaria BM retains robust erythropoietic capacity, in stark contrast to the adipocyte‐rich and functionally impaired long bone marrow in diabetic mice.

**FIGURE 5:**
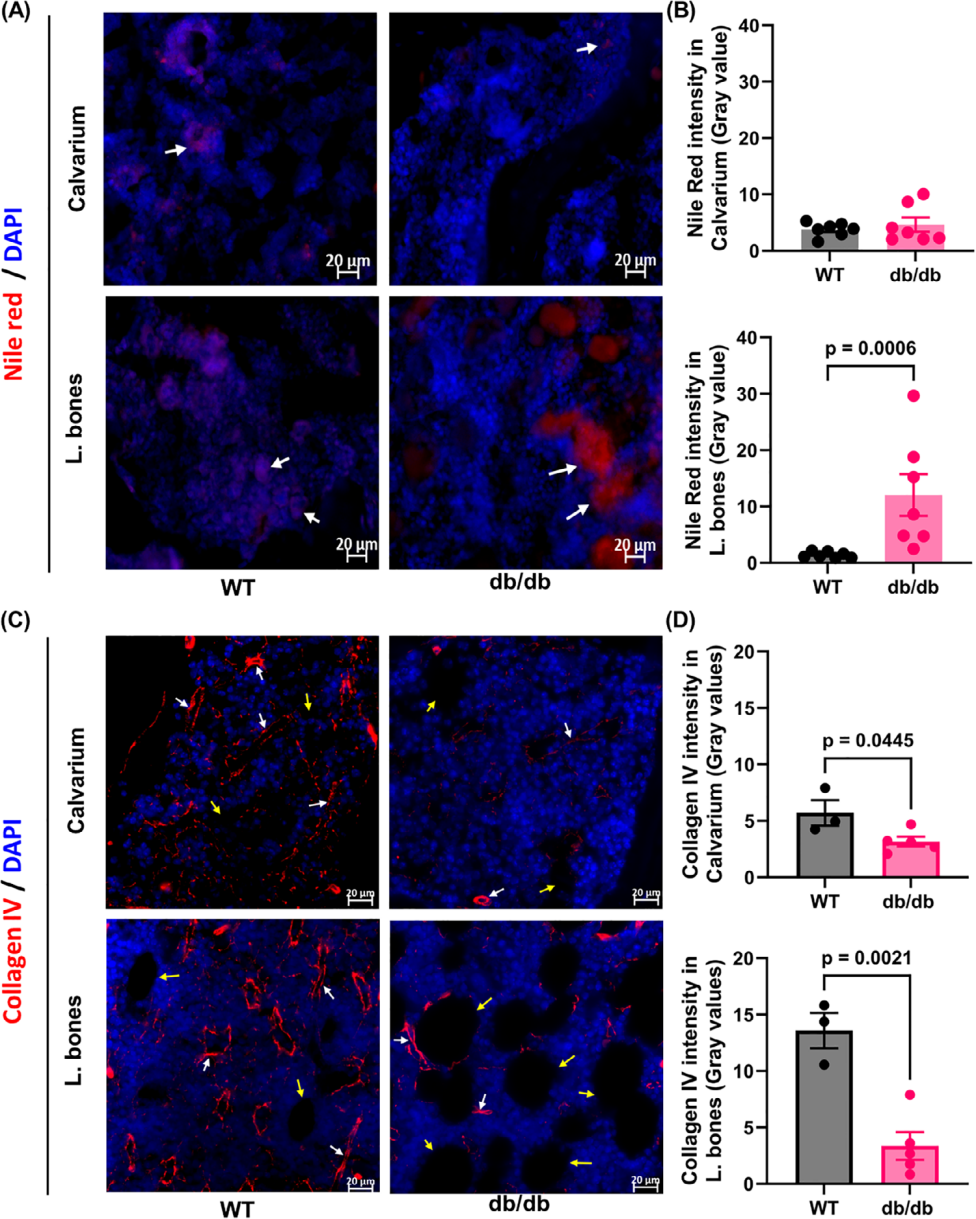
Lipid/Fat content and changes in the vasculature of the calvarium and tibial compartments. (A, B) Lipid accumulation in bone marrow compartments. Representative images (A) and mean fluorescence intensity (B) quantification of Nile Red staining in calvaria and long bones from WT and db/db mice, indicating differences in lipid content between genotypes and marrow sites. (C, D) Vascular structure assessment via Collagen IV staining. (C) Representative images and (D) mean fluorescence intensity quantification of Collagen IV staining in calvaria and long bones of WT and db/db mice, reflecting changes in vascularity across bone marrow niches. n = 3–7 mice per group.

Nile Red, a lipophilic dye, stains neutral lipids and hydrophobic regions. In db/db mice, Nile Red fluorescence was elevated in long bones but unchanged in the calvarium (Figure 5A,B), indicating increased lipid accumulation in long bones only. At 12 months, both bone compartments showed reduced vascular density compared to controls, as demonstrated with decreased collagen IV intensity (Figure 5C,D), with a more pronounced decline in long bones. These findings point to a slower rate of vascular degeneration within the calvaria bone marrow compared to other skeletal sites. This observation prompted the question: what intrinsic or extrinsic factors confer this apparent protection to the calvarium?

### 2.4 CSF Contains Elevated Levels of Oxysterols That Potentially Protect the Calvarium BM Compartment

**FIGURE 6:**
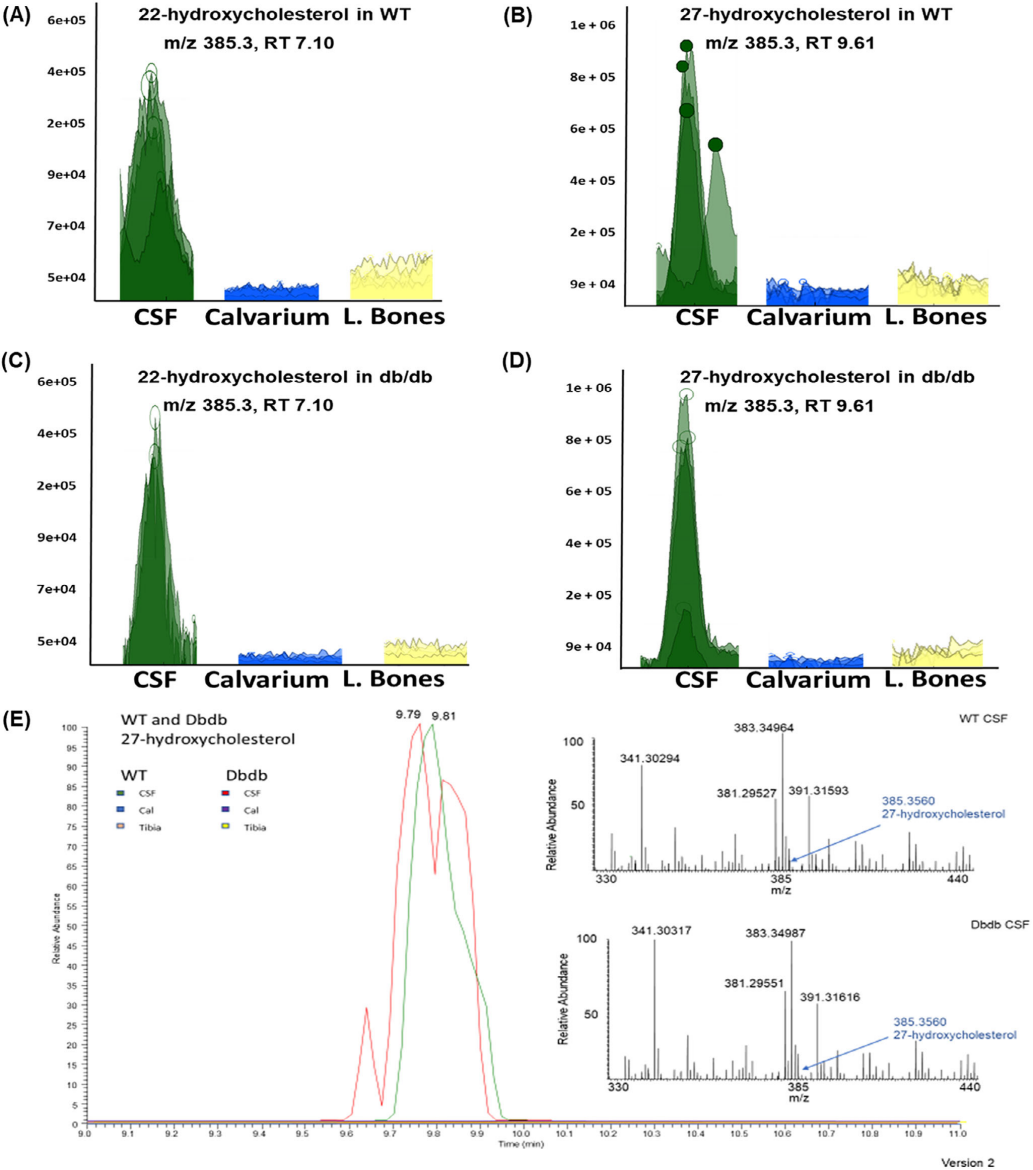
High‐resolution/accurate mass LC‐nESI‐MS analysis of oxysterols in CSF and BM compartments of calvaria and long bones. (A–D) Base peak chromatograms showing masses corresponding to oxysterol ions ([M–H_2_O]⁺ and [M–2H_2_O]⁺) obtained via reverse‐phase liquid chromatography for 22‐hydroxycholesterol (A, C) and 27‐hydroxycholesterol (B, D) in WT and db/db mice. (E) Zoomed view of the 27‐hydroxycholesterol peak in CSF from control and diabetic mice. The inset displays the LTQ‐Orbitrap Velos mass spectrum at the corresponding retention time, highlighting mass‐to‐charge (m/z) values for 27‐hydroxycholesterol ions in WT and db/db CSF samples. n = 3–7 mice per group.

To assess the composition of the CSF, we performed LC‐nESI‐MS analysis of CSF and BM supernatants from WT and db/db mice. In 12‐month WT mice, CSF contained high levels of 22‐OHC and 27‐OHC (Figure 6A,B), with lower levels in BM. Notably, despite reduced plasma oxysterols in db/db mice, CSF levels of 22‐OHC and 27‐OHC remained elevated (Figure 6C,D), suggesting that calvarial BM retains access to these bioactive lipids, unlike long bones.

To validate these findings in humans, we analyzed CSF from individuals with and without T2D (T2D defined by HbA1c >5.6%) using untargeted metabolomics data from a separate study on ideopathic intracranial hypertension (IIH). We identified 7α‐hydroxy‐3‐oxo‐4‐cholestenoic acid (7‐HOCA), a metabolite of 27‐OHC, in both diabetic and non‐diabetic individuals. As shown in Table 1, 7‐HOCA levels were similar across groups, suggesting that beneficial CSF‐derived metabolites are maintained in diabetes, and this may support functional hematopoiesis in the calvarium.

**TABLE 1:** 7‐HOCA levels in the diabetic individuals with and without IIH in comparison of normal healthy individual.

Variable	Control individuals	Diabetic individuals (HbA1c > 5.6)	
		Without IIH	With IIH
Number of individuals in each cohorts	7	6	8
Sex			
Male	0	5	2
Female	7	1	6
Age Range			
<30 year	0	0	1
30–50 years	5	1	6
50–70 years	2	2	1
>70 years	0	3	0
BMI			
30–40	4	5	2
40–50	3	1	3
>50	0	0	3
HbA1c			
<5.6%	7	0	0
>5.6%	0	6	8
			
Other Diagnosis			
IIH	6	0	8
Hydrocephalus	1	3	0
			
7‐HOCA levels	0.6382	0.9956	1.1787

### 2.5 Preferential Recruitment of Hematopoietic Cells From the Calvarium Into the Retina Following Acute Ischemia/Reperfusion (I/R) Injury

**FIGURE 7:**
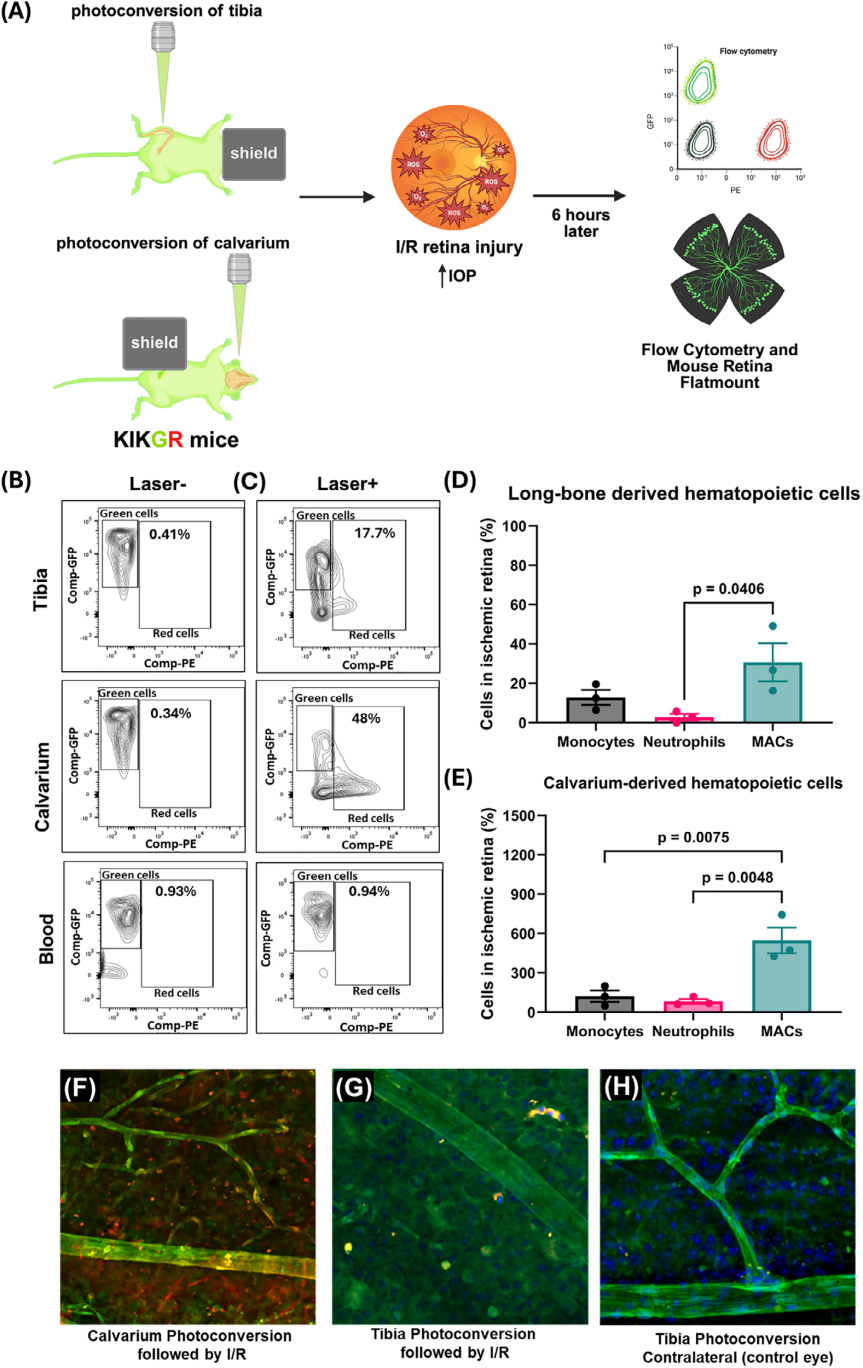
In vivo BM cell labelling by photoconverting calvaria and tibia marrow of KIKGR mice for cell tracking. (A) Schematic of the experimental design for tracking bone marrow (BM) cell recruitment to the injured retina. KikGR mice underwent photoconversion of either the tibial or calvarial BM compartments via targeted laser exposure. Retinal ischemia‐reperfusion (I/R) injury was induced immediately after, and tissues were harvested for flow cytometry 6 h post‐injury. (B, C) Representative flow cytometry plots showing proportions of live photoconverted (PE⁺, red) CD45⁺ cells in the tibia, calvarium, and peripheral blood of uninjured control mice before (B) and immediately after (C) photoconversion. (D, E) Quantification of BM‐derived cell recruitment to the retina following I/R injury. Bar graphs show the normalized relative contributions of photoconverted BM cells from the calvarium (D) and tibia (E) to the retina as monocytes, neutrophils, and MACs. (F) Retinal flat mount from a KikGR mouse with calvarium photoconversion followed by I/R injury, showing red photoconverted cells recruited to the retina. (G) Retinal flat mount from a KikGR mouse with tibia photoconversion followed by I/R injury, showing fewer photoconverted cells (yellow/orange), indicating partial conversion and reduced recruitment. (H) Retinal flat mount from the contralateral eye of a KikGR mouse with tibia photoconversion but no I/R injury, showing absence of photoconverted cells confirming injury‐dependent recruitment seen in f and g. n = 3 mice per group; data representative of two independent experiments.

In earlier work, we showed that subthreshold retinal phototherapy recruits BM‐derived cells to the retina and RPE [25] originally assuming mobilization from long bones. Given the presence of skull channels, we re‐examined this using the I/R retinal injury model in *Kikume Green‐Red mice*. Kikume Green‐Red mice express a unique protein, Kikume Green‐Red, which is a photoconvertible fluorescent protein originally engineered from a stony coral (Favia favus). It fluoresces green under normal conditions, but upon exposure to violet or UV light (∼405 nm). It undergoes an irreversible conformational change and fluoresces as red. Either the calvarium or tibia was photoconverted to “label” BM cells from green to red (Figure 7A). The calvarium showed greater conversion than the tibia due to its thin, flat structure (Figure 7C). Following I/R injury, MACs were the dominant mobilized population, regardless of the photoconversion site (Figures 7D,E; S6 Gating strategy and Figure S7A–C). Notably, MAC recruitment from the calvarium was ∼20‐fold higher than from the tibia (Figure 7E). Flow cytometry of WT BM showed similar proportions of MACs and neutrophils between compartments, suggesting the difference was not due to cell composition (Figure S5D–F). Flat‐mounted retinas confirmed more photoactivated cells following calvarium photoconversion (Figure 7F) than tibia (Figure 7G), with no activation in contralateral controls (Figure 7H). These results indicate that the calvarium is a major source of MACs and neutrophils mobilized to the injured retina. These findings reveal that the calvarium serves as a more active and accessible reservoir for myeloid cell mobilization to the injured retina than long bones, likely due to its anatomical structure and proximity to the CNS.

## 3 Discussion

Our murine data, along with secondary analysis of CSF from IIH patients, support that diabetic CSF retains sufficient levels of protective oxysterols to sustain physiologic haematopoiesis, highlighting the role of skull channels in neuroimmune communication across species [1, 5, 26, 27, 28]. The meninges are connected to the overlying calvaria bone marrow via osseous channels containing blood vessels that link meningeal circulation to the BM sinusoidal network. These channels enable bidirectional trafficking, allowing hematopoietic cells to migrate into the meninges while permitting CSF to penetrate and interact with bone marrow compartments.

This unique access to CSF, rich in neurotrophic and growth factors, may support haematopoiesis in calvaria and vertebral BM. Contrary to the prevailing view that BM niches deteriorate with age [29] or diabetes, our findings show that the calvarium maintains its structural integrity, channel density, and robust haematopoietic activity during aging and chronic metabolic disease. To our knowledge, this study provides the first detailed characterization of skull channel architecture in the frontal, parietal, and occipital regions of WT and diabetic mice. We show that chronic diabetes does not compromise calvaria structure, and despite prolonged metabolic dysregulation in db/db mice, calvaria hematopoiesis remains intact with robust erythropoiesis and no excessive myelopoiesis, in contrast to the diabetic long bones [11]. While not observed in our study, altered skull channel dimensions may have functional consequences. These channels connect calvaria marrow to the meninges and act as conduits for myeloid cell trafficking during neuroinflammation and injury. A reduction in channel length or diameter could impair the efficiency of immune cell migration from BM to the CNS, potentially delaying or limiting local immune responses. Conversely, changes in channel architecture might influence CSF exchange and pressure dynamics, as these channels form part of the interface between BM and meningeal vasculature. Although direct evidence linking channel size to CSF flow is limited, their proximity to dural sinuses suggests a potential role in fluid and solute clearance.

Importantly, we demonstrate for the first time that calvaria BM contributes reparative immune cells to the retina following acute injury. While anatomical proximity to the eye likely facilitates this response, the optic nerve like the calvarium is also surrounded by CSF and directly connected to the neuroimmune axis [30]. Previously, we showed that MACs support postnatal retinal development [31], further reinforcing the link between calvaria haematopoiesis and ocular health.

We identified key differences between calvaria and long BM compartments in aging and diabetes. Unlike long bones, the calvarium does not accumulate fat with age or metabolic disease. Adipogenesis in long bones contributes to myelopoiesis, a hallmark of diabetic (long bone) marrow dysfunction, whereas the calvarium remains resistant to this shift. In db/db mice, calvaria BM maintains robust erythropoiesis, while long bones develop fatty (“yellow”) marrow and skew toward myeloid‐biased haematopoiesis. Previous studies have shown that diabetes promotes adipocyte accumulation in long bones by driving mesenchymal stem cells toward adipogenic rather than osteogenic differentiation, impairing hematopoietic stem cell function [32, 33]. However, these studies focused exclusively on long bones. Our findings caution against generalizing these effects to all BM compartments, particularly the calvarium. Instead of responding to metabolic stress with adipogenesis, the calvarium compensates with enhanced erythropoiesis. It is plausible that as hematopoiesis deteriorates in long bones, the calvarium assumes a compensatory role, supporting systemic hematopoiesis and supplying reparative cells when other compartments fail [34].

Previous studies have shown that streptozotocin‐induced type 1 diabetes (T1D), as well as T2D in db/db mice and rats, leads to denervation in long bones, promoting myelopoiesis [35, 36, 37, 38, 39, 40, 41]. In contrast, Ferraro et al. reported neurogenesis in the calvarium of diabetic mice, suggesting a compartment‐specific response to diabetes [42]. Our current findings support this distinction, highlighting the calvarium's unique access to CSF, which delivers neurotrophic and growth factors that sustain both hematopoiesis and neurogenesis. Diabetic mice exhibit neuropathy within long bones, which disrupts neurovascular regulation of the bone marrow niche and skews hematopoiesis toward inflammatory myelopoiesis. This neuropathic damage is driven by hyperglycemia‐induced oxidative stress, advanced glycation end‐products, and loss of sympathetic innervation, leading to impaired β3‐adrenergic signaling and reduced CXCL12 gradients that normally maintain hematopoietic stem cell quiescence [11]. In contrast, the calvarial marrow appears relatively protected, likely due to elevated neuroprotective oxysterols that activate liver X receptor (LXR) pathways, promote cholesterol efflux, suppress NF‐κB‐mediated inflammation [43], and support neuronal survival signaling. These mechanisms may preserve perivascular innervation and vascular integrity in the calvarium, maintaining hematopoietic homeostasis compared to the neuropathy‐prone long bone marrow.

The skull marrow represents a neuroimmune hub. These factors collectively influence immune cell trafficking to CNS, shape bone remodeling and marrow niche stability, and provide metabolic and signaling cues that can either amplify or resolve neuroinflammation. Brain‐Derived Neurotrophic Factor (BNDF) promotes survival and differentiation of neurons but also influences hematopoietic stem cells (HSCs) and immune cells in the marrow and can modulate sympathetic innervation of bone marrow, affecting mobilization of progenitors [44]. Glial cell line‐Derived Neurotrophic Factor (GDNF) supports neuronal health and may indirectly regulate the marrow niche via Schwann cells and perivascular glia, and emerging evidence suggests neurotrophic factors can alter osteoblast activity, impacting marrow microenvironment [45]. IL‐10 suppresses pro‐inflammatory cytokine production in marrow‐resident macrophages and may reduce neuroinflammation signals transmitted through skull channels [46]. TGF‐β is critical for HSC quiescence and niche maintenance but also influences osteogenesis and bone remodeling, shaping the diploë and marrow cavity [47]. Thus, CSF‐derived factors may enhance HSC stress resistance and modulate immune cell phenotype toward regulatory profiles.

Maintaining MACs is critical for retinal vascular protection. While long bones rapidly succumb to diabetes‐induced damage losing MACs and accumulating proinflammatory myeloid cells, the calvarium preserves physiological hematopoiesis. We postulate that in individuals with longstanding diabetes, vascular complications such as diabetic retinopathy may arise when hematopoiesis fails in the long bones, and the calvarium can no longer compensate by supplying vascular‐protective cells like MACs. Based on our findings, we propose that the 20‐fold higher recruitment of MACs reflects an anatomical advantage of the calvarium compared to long bones. Signals from the injured retina likely drive this recruitment through cytokines such as CXCL12, as MACs express their cognate receptor, CXCR4. The calvarium contains ossified channels that directly connect the diploë, the spongy bone layer housing red marrow, to the meninges and dural sinuses. These channels, which contain vessels, provide short, low‐resistance routes for myeloid cells to enter circulation or migrate to the retina and CNS. In contrast, long bones require cells to traverse more complex sinusoidal networks. Previous studies have shown that the calvaria niche is exposed to CSF‐borne antigens and inflammatory signals, enabling rapid egress of myeloid cells during CNS injury or infection, a signaling pathway less pronounced in long bones due to their distance from the CNS. Here, we demonstrate that rapid MAC egress also occurs in response to retinal injury and that CXCL12 levels differ between calvaria and tibial marrow. Thus, the calvarium functions as a myeloid reservoir for rapid immune responses in the CNS and meninges, whereas long bones primarily support systemic hematopoiesis. The calvarium's anatomical shortcuts, CNS proximity, unique chemokine dynamics, and specialized immune role make it a preferential source for hematopoietic cell release under stress or inflammatory conditions of the eye and brain.

Micro‐CT analysis was used to compare structural differences between the calvarium and long bones. In diabetic mice, trabecular thickness increased in the calvarium but decreased in long bones, relative to age‐matched controls. T2D is typically associated with osteoporosis due to an imbalance between osteoblast and osteoclast activity, favoring bone resorption and impairing bone formation [48]. These changes were evident in long bones but not in the calvarium. Although T2D negatively affects bone mass and quality, its impact is less severe than in T1D [49, 50].

Diabetes causes microangiopathy in peripheral tissues, including BM, and is characterized by endothelial dysfunction, increased vascular permeability, and capillary loss [51, 52]. Both calvarial and long bone marrow showed reduced vascular density in diabetic mice compared to controls; however, the reduction in the calvarium was approximately fourfold less than in long bones. In chronic diabetes, the calvarium exhibited increased trabecular thickness, lower adiposity, slower vascular degeneration, and preserved HSPC number and function, highlighting its resilience compared to long bones.

Our studies show that both WT and T2D mice have elevated levels of the oxysterols 22‐OHC and 27‐OHC in their CSF, while plasma levels remain low [35]. Elevated CSF oxysterol levels in db/db mice, despite reduced plasma concentrations, strongly support a CNS‐specific mechanism. The brain possesses sterol transport pathways (e.g., ABC transporters) that can promote selective retention or influx of oxysterols into the CSF [53]. Under metabolic stress, brain cholesterol metabolism is known to increase. Thus, this discrepancy underscores a physiological adaptation within the CNS and highlights oxysterols as potential mediators of neuroprotection.

This also suggests that long bones, which rely on blood perfusion, are exposed to reduced levels of these protective lipids, potentially contributing to the compartment‐specific differences observed. Hematopoietic cells express LXRs, which require oxysterols as natural ligands for proper function. Previously, we demonstrated that N‐dimethyl‐3β‐hydroxycholenamide (DMHCA), an LXR agonist, enhances erythropoiesis and MAC production [35], further supporting the role of oxysterols in BM protection. Interestingly, the beneficial effects of DMHCA on diabetic long bone hematopoiesis, such as increased erythrocyte generation and reduced inflammatory monocytes mirror the untreated calvarium's response in diabetic mice. This highlights the calvarium's unique resilience and access to CSF‐derived oxysterols, which may sustain physiological hematopoiesis despite chronic metabolic stress.

BM‐derived cells contribute to tissue repair following injury, including in the retina [37, 41, 54]. The calvarial BM, anatomically connected to the CNS via skull channels, serves as a rapid‐response immune reservoir in both mice and humans, particularly following brain injury such as stroke [1]. We investigated whether this compartment also responds to retinal injury. In our I/R model, retinal damage occurs due to hypoxia and oxidative stress during reperfusion [12]. Following I/R injury, the calvarium BM contributed significantly more neutrophils and MACs to the retina than long bones. Neutrophils act as first responders and promote neuronal survival through paracrine signaling [55], supporting the calvarium as a primary source of reparative immune cells in acute retinal injury.

Importantly, the CSF communicates directly with the calvarium BM and surrounds the optic nerve head via the subarachnoid space [56]. Thus, inflammatory signals released by the injured retina, such as TNFα and IFNs may travel through the CSF to the calvarium BM, triggering a localized hematopoietic response [57, 58]. This route likely enables faster activation than systemic circulation, which would engage both calvarium and long bone compartments simultaneously. Future studies will explore this potential CSF‐mediated communication pathway between the retina and calvarial BM.

Our study has several limitations. First, we did not assess whether MACs recruited from the calvarium are functionally distinct from those originating in long bones. Second, we relied on a single diabetes model, the db/db mouse. Although the high‐fat diet (HFD) model [59, 60] is commonly used, it has limitations such as variable responses to dietary intervention. Moreover, our focus was on oxysterol composition in CSF and BM. Using a diet‐induced model could confound these measurements, as we could not distinguish whether changes in oxysterols detected in the calvarium were due to the high‐fat diet or the calvarium's intrinsic capacity to maintain beneficial oxysterols. Third, our studies do not establish the causal significance of oxysterols in the CSF; however, the lack of knockout models that are specific for oxysterols limits our interrogation of this hypothesis. In future studies, we are intended to investigate the direct impact of 22‐OHC, 27‐OHC, and 7‐HOCA on BM populations derived from diabetic mice. These studies would allow us to ascertain the regenerative potential of these oxysterols in correction of BM cell dysfunction and the specific signaling pathways they restore.

## 4 Conclusion

In conclusion, we provide the first comprehensive characterization of skull channel architecture in the frontal, parietal, and occipital regions of WT and diabetic mice across two time points. Remarkably, this complex network remains structurally intact in chronic diabetes, preserving communication between the calvaria BM and CSF. This direct CSF access supports the function and proliferative potential of hematopoietic cells in the calvarium. Unlike systemic circulation, CSF‐mediated signaling enables rapid, localized BM responses to CNS and retinal injury, offering an evolutionary advantage. We show that CSF in both diabetic and non‐diabetic mice contains elevated levels of protective oxysterols, 22‐OHC, 27‐OHC, and 7‐HOCA, which are maintained in diabetes, unlike their plasma levels. This unique exposure allows the calvarium BM to sustain physiological hematopoiesis despite chronic metabolic stress. Collectively, our findings highlight the calvarium BM as a specialized hematopoietic niche, distinct from long bones, with preserved architecture, vascular integrity, and immune function. The bidirectional exchange between CSF and calvaria BM supports its role in development, homeostasis, and defense against CNS and ocular injury, positioning it as a critical neuroimmune interface.

## 5 Experimental Section

### 5.1 Ethical Statement

The human samples used in this study represent a secondary analysis of the initial data [61]. Briefly, CSF was collected from 25 patients with a known diagnosis of Idiopathic Intracranial Hypertension (IIH) undergoing CSF diversion therapy at the Ohio State University Wexner Medical Center (IRB #2018H0288 and #2022H0157). Consent was obtained for collection of CSF for bio‐banking. The control cohort consisted of 22 patients who underwent CSF shunting or CSF analysis for conditions other than IIH. All patients were clinically obese with a BMI greater or equal to 30, and when possible HbA1c was recorded to determine diabetic status [61]. All procedures adhered to institutional ethical guidelines and the principles outlined in the Declaration of Helsinki.

### 5.2 Metabolomic Analysis of Human CSF

Metabolomic analysis was performed by Metabolon, Inc. using untargeted mass spectrometry. Untargeted global metabolomics was conducted using UPLC‐MS/MS, leveraging a comprehensive library of over 5400 annotated metabolites and an additional 7000 unidentified compounds, to evaluate functional differences among CSF samples [61].

### 5.3 Animals

Animal work described was approved by the Institutional Animal Care and the Use Committee at the University of Alabama at Birmingham (APNs 21261, 21291, 21802, and 22616). The studies adhere to the Association for Research in Vision and Ophthalmology Statement on the Use of Animals in Ophthalmic and Visual Research. Mice were housed in a standard laboratory environment under a 12 h light/dark cycle.

### 5.4 Isolation of BM Cells From Long Bones and Calvarium and Flow Cytometry

Briefly, animals were euthanized, long bones (tibia) and calvaria were carefully dissected from the legs and head, respectively. Bones were sectioned using surgical scissors and gently crushed with a mortar and pestle for up to 1 min, repeated three times in 1 mL of FACS buffer. The resulting bone fragments were filtered through a 40 µm strainer into a 50 mL conical tube and centrifuged at 350 × *g* for 5 min. Cell pellets were resuspended, and red blood cells were lysed using 1–2 mL of ACK lysis buffer. The cells were then washed twice with FACS buffer and resuspended for downstream applications.

### 5.5 Flow Cytometry

BM and peripheral blood and single cell suspensions of the retina were prepared for flow cytometry as previously published [31, 62]. After washing with FACs buffer, single cell suspensions were incubated with primary antibody cocktails for 45 min in the dark. All antibodies used are described in Table S1.

### 5.6 X‐Ray Micro‐CT Scanning of Calvarium and Femur

Calvarium and femur were removed from db/db mice and age matched controls, and skin and soft tissue carefully removed. Bones were fixed in 4% PFA at 40°C overnight, then washed and transferred into PBS. Samples for X‐ray micro‐CT were contained in a plastic tube, and scans were conducted with a Zeiss X‐radia Versa 620 X‐ray microscope. For micro‐CT analysis, measurements were performed in anatomically identical regions of each mouse's paired bones, the calvarium and femur, to ensure consistency and comparability across samples. Regions of interest (ROIs) were defined using standardized anatomical landmarks, and all scans were acquired under uniform imaging parameters to minimize variability. Of each calvarium or long bone sample, three scans were conducted: a whole‐sample scan at 16.8 µm (calvarium) or 19 µm (femur), and two region‐of‐interest (ROI) scans at 2 µm voxel size. All scans were acquired with a source energy of 60 kV, 6.5 W, and using a LE1 beam filter, with exposure times between 1 s (full samples) and 2 or 7 s (ROI of femur or calvarium). The projection data was reconstructed using Zeiss Scout and Scan Reconstructor 16.7 with the Deep Recon option for image improvement and noise suppression. Segmentation and visualization of the data was conducted with Drishti [63] and Drishti Paint [64]. Quantitative measurements of bone and channel dimensions were performed using BoneJ [65] plugins for Fiji [66].

### 5.7 Bone Marrow Compartment Photoconversion

KIKGR transgenic mice were anesthetized with ketamine (80 mg/kg) and xylazine (15 mg/kg). Fur over the skull and tibia was shaved, and the skin disinfected with 70% ethanol. For calvaria exposure, a vertical incision was made along the sagittal suture, extending anteriorly to the frontal calvarium and posteriorly to the occipital calvarium. Overlying membranes were gently exercised to allow flexible skin retraction. The skin flaps were held apart with tape to fully expose the calvarium. Ocular lubricant was applied, and the anterior portion of the head, including both eyes, was covered with aluminum foil secured with tape. To prevent unintended photoconversion, the rest of the body was also covered with foil.

For tibial exposure, a small incision was made along the medial aspect of the tibia, extending from the patella to the distal tibia. Under a dissecting microscope, muscle tissue was carefully removed to expose the bone, and the legs were stabilized with tape. The remainder of the body was covered with aluminum foil to prevent off‐target photoconversion.

Photoconversion of bone marrow cells was performed by illuminating the anterior and posterior halves of the exposed bones for 3 min each using a 405 nm laser (Dymax Bluewave QX4 V2.0, Dymax Corporation, Torrington, CT) fitted with a 5 mm‐diameter focusing lens at 75% power. The lens was positioned 5 cm from the bone surface, for a total exposure time of 6 min. Following photoconversion, skin incisions were closed using Vetbond tissue adhesive.

### 5.8 Acute Retinal Ischemia/Reperfusion Injury (I/R)

Retinal I/R procedure was modified as previously published [67, 68]. Under deep isoflurane anesthesia, right eyes were cannulated with a 30‐gauge needle connected to a sterile saline infusion bag to elevate the IOP (80–90 mmHg) for 1 h. Whitening of the iris and loss of red reflex confirmed retinal ischemia. After 1 h of ischemia, the needle was removed, and the eyes were allowed to reperfuse. Mice were euthanized after 6 h [68].

### 5.9 CXCL12 ELISA

Samples were analysed for CXCL12 levels using the Mouse CXCL12/SDF‐1 DuoSet ELISA kit (R&D Systems, Cat# DY460) according to manufacturer's instructions.

### 5.10 Colony‐Forming Unit (CFU) Assay

CFU assay was performed as previously published [35].

### 5.11 Immunofluorescence Staining

Immunohistochemical procedures were modified according to our published protocols [69]. Images were obtained with a 40X objective lens and quantified using ImageJ. Briefly in each image, individual color channels were separated using the “Split Channels” function. The channel of interest was converted to 8‐bit grayscale, and the image scale was calibrated using a scale bar. To isolate the fluorescent signal, an appropriate threshold was applied under “Adjust Threshold,” ensuring accurate segmentation of positive staining. The average intensity (darkness/color) of pixels within a region of interest (ROI) was then identified and added to the ROI manager using the “Analyze Particles” tool. All the measurements were performed by selecting the mean gray value following background correction (measuring fluorescence in regions without staining). All measurements were exported for statistical analysis, and identical imaging settings (laser power, exposure, and gain) were maintained across samples to ensure comparability. In IHC quantification, the mean gray value represents the average pixel intensity. In physical terms, this is analogous to measuring the concentration of the substance (the protein), not the total amount of the substance.

### 5.12 Nile Red and Collagen IV Staining

Decalcified bones were incubated in 30% sucrose in PBS for 48 h at 4°C, embedded in optimal cutting temperature (OCT) compound, immediately frozen on dry ice, and stored at −80°C. Cryosections were prepared and stained with 5 µg/mL Nile Red solution at room temperature for 15 min, followed by washing and counterstaining with DAPI for 10 min. Immunofluorescence staining for collagen IV was carried out as described previously [31, 70]. After a final wash, sections were mounted for imaging.

### 5.13 Oxysterols in CSF and BM Supernatants

Cerebrospinal fluid (CSF) and bone marrow (BM) supernatants (10 µL each) were thawed on ice and subjected to lipid extraction using the tert‐butyl methyl ether (MTBE) method, as previously described [71]. Briefly, samples were mixed with methanol and MTBE in a biphasic extraction system to efficiently partition sterols into the organic phase. After phase separation, the organic layer was collected, dried under nitrogen, and reconstituted in an appropriate solvent for analysis. Oxysterol species were quantified using liquid chromatography‐tandem mass spectrometry (LC‐MS/MS) with multiple reaction monitoring (MRM) for specific oxysterols (e.g., 24S‐hydroxycholesterol, 25‐hydroxycholesterol, 27‐hydroxycholesterol). Internal standards (deuterated oxysterols) were included to ensure accurate quantification. Data were normalized to sample volume and expressed as ng/mL [72].

### 5.14 Statistical Analysis

Statistical analyses were performed using GraphPad Prism v9.1 software. Data are reported as mean ± SD. All data were assessed for adherence to normal distribution by the Shapiro‐Wilks normality test. Data that conformed to normal distribution were analysed by unpaired Student's t‐test, and data that failed to meet the assumptions of normality were analysed using unpaired Mann‐Whitney and one‐tailed test. *p*‐values less than 0.05 were considered statistically significant.

## Author Contributions

B.A‐B., S.L.C. and J.G.B. performed majority of the experiments and analysed data. B.A‐B., S.L.C., M.E.B., J.V.B., and M.B.G. analysed data and wrote the manuscript. R.P., Y.A‐A., R.F.R., J.L.F., A.R., D.S., A.L., T.A.L., C.M.S., and J.V.B. performed a subset of experiments and analysed corresponding data. B.A.B and M.B.G designed and directed the study. K.L.T. and M.B. provided metabolomics data from human CSF and revised the manuscript. All authors reviewed the manuscript and provided final approval for submission.

## Funding

Work from our lab is funded by the National Institutes of Health grants, R01EY012601 to JVB and MBG, R01EY028858, R01EY028037, R01EY025383, R01EY032753, and EY033620 to M.B.G and Research to Prevent Blindness unrestricted grant awarded to Department of Ophthalmology and Visual Sciences at UAB. Our study was also supported by the UAB Core Grant for Vision Research, P30 EY003039 from the National Eye Institute. KLT and MB data from IIH patients was supported by a grant from The Ohio State College of Medicine Dean's Intramural Funding Discovery mechanism.

## Conflicts of Interest

The authors declare no conflicts of interest.

## Supporting information




**Supporting File 1**: advs73436‐sup‐0001‐Figures.pptx.

**Supporting File 2**: advs73436‐sup‐0002‐Table S1.docx.

**Supporting File 3**: advs73436‐sup‐0003‐Video S1.pptx.

**Supporting File 4**: advs73436‐sup‐0004‐Video S2.pptx.

**Supporting File 5**: advs73436‐sup‐0005‐Video S3.pptx.

## Data Availability

The data that support the findings of this study are available from the corresponding author upon reasonable request.

## References

[1] F. Herisson , V. Frodermann , G. Courties , et al., “Direct Vascular Channels Connect Skull Bone Marrow and the Brain Surface Enabling Myeloid Cell Migration,” Nature Neuroscience 21, no. 9 (2018): 1209–1217, 10.1038/s41593-018-0213-2. 30150661 PMC6148759

[2] C. Dobersalske , L. Rauschenbach , Y. Hua , et al., “Cranioencephalic Functional Lymphoid Units in Glioblastoma,” Nature Medicine 30, no. 10 (2024): 2947–2956. 10.1038/s41591-024-03152-x PMC11485206 39085419

[3] R. Cai , C. Pan , A. Ghasemigharagoz , et al., “Panoptic Imaging of Transparent Mice Reveals Whole‐body Neuronal Projections and Skull–meninges Connections,” Nature Neuroscience 22, no. 2 (2019): 317–327, 10.1038/s41593-018-0301-3. 30598527 PMC6494982

[4] A. Cugurra , T. Mamuladze , J. Rustenhoven , et al., “Skull and Vertebral Bone Marrow Are Myeloid Cell Reservoirs for the Meninges and CNS Parenchyma,” Science 373, no. 6553 (2021): abf7844. 10.1126/science.abf7844 PMC8863069 34083447

[5] J. A. Mazzitelli , L. C. D. Smyth , K. A. Cross , et al., “Cerebrospinal Fluid Regulates Skull Bone Marrow Niches via Direct Access through Dural Channels,” Nature Neuroscience 25, no. 5 (2022): 555–560. 35301477 10.1038/s41593-022-01029-1 PMC9081158

[6] X. J. Tong , G. Akdemir , M. Wadhwa , A. S. Verkman , and A. J. Smith , “Large Molecules from the Cerebrospinal Fluid Enter the Optic Nerve but Not the Retina of Mice,” Fluids Barriers CNS 21, no. 1 (2024): 1. 38178155 10.1186/s12987-023-00506-4 PMC10768282

[7] R. A. Shahror , C. A. Morris , A. A. Mohammed , et al., “Role of Myeloid Cells in Ischemic Retinopathies: Recent Advances and Unanswered Questions,” J Neuroinflammation 21, no. 1 (2024): 65. 38454477 10.1186/s12974-024-03058-y PMC10918977

[8] G. P. Fadini , F. Ferraro , F. Quaini , T. Asahara , and P. Madeddu , “Concise Review: Diabetes, the Bone Marrow Niche, and Impaired Vascular Regeneration,” Stem Cells Translational Medicine 3, no. 8 (2014): 949–957, 10.5966/sctm.2014-0052. 24944206 PMC4116251

[9] P. Ramalingam , M. C. Gutkin , M. G. Poulos , et al., “Restoring Bone Marrow Niche Function Rejuvenates Aged Hematopoietic Stem Cells by Reactivating the DNA Damage Response,” Nature Communications 14, no. 1 (2023): 2018. 10.1038/s41467-023-37783-4 PMC10086043 37037837

[10] S. K. Pasupuleti , B. Ramdas , S. S. Burns , et al., “Obesity‐induced Inflammation Exacerbates Clonal Hematopoiesis,” Journal of Clinical Investigation 133, no. 11 (2023): e163968, 10.1172/JCI163968. 37071471 PMC10231999

[11] J. V. Busik , M. Tikhonenko , A. Bhatwadekar , et al., “Diabetic Retinopathy is Associated with Bone Marrow Neuropathy and a Depressed Peripheral Clock,” Journal of Experimental Medicine 206, no. 13 (2009): 2897–2906, 10.1084/jem.20090889. 19934019 PMC2806461

[12] A. Tsung , R. A. Hoffman , K. Izuishi , et al., “Hepatic Ischemia/Reperfusion Injury Involves Functional TLR4 Signaling in Nonparenchymal Cells,” The Journal of Immunology 175, no. 11 (2005): 7661–7668, 10.4049/jimmunol.175.11.7661. 16301676

[13] Y. Duan , R. Prasad , D. Feng , et al., “Bone Marrow‐Derived Cells Restore Functional Integrity of the Gut Epithelial and Vascular Barriers in a Model of Diabetes and ACE2 Deficiency,” Circulation Research 125, no. 11 (2019): 969–988, 10.1161/CIRCRESAHA.119.315743. 31610731 PMC7056264

[14] C. Urbich , A. Aicher , C. Heeschen , et al., “Soluble Factors Released by Endothelial Progenitor Cells Promote Migration of Endothelial Cells and Cardiac Resident Progenitor Cells,” Journal of Molecular and Cellular Cardiology 39, no. 5 (2005): 733–742, 10.1016/j.yjmcc.2005.07.003. 16199052

[15] E. Beli , J. M. Dominguez , P. Hu , et al., “CX3CR1 deficiency Accelerates the Development of Retinopathy in a Rodent Model of Type 1 Diabetes,” Journal of Molecular Medicine 94, no. 11 (2016): 1255–1265, 10.1007/s00109-016-1433-0. 27344677 PMC5071129

[16] Y. P. Jarajapu , S. Hazra , M. Segal , et al., “Vasoreparative Dysfunction of CD34+ Cells in Diabetic Individuals Involves Hypoxic Desensitization and Impaired Autocrine/Paracrine Mechanisms,” PLoS ONE 9, no. 4 (2014): 93965. 10.1371/journal.pone.0093965 PMC3979711 24713821

[17] D. W. Russell , “Oxysterol Biosynthetic Enzymes,” Biochimica Et Biophysica Acta (BBA)—Molecular and Cell Biology of Lipids 1529, no. 1‐3 (2000): 126–135, 10.1016/S1388-1981(00)00142-6. 11111082

[18] D. R. Bauman , A. D. Bitmansour , J. G. McDonald , B. M. Thompson , G. Liang , and D. W. Russell , “25‐Hydroxycholesterol Secreted by Macrophages in Response to Toll‐Like Receptor Activation Suppresses Immunoglobulin A Production,” Proceedings of the National Academy of Sciences 106, no. 39 (2009): 16764–16769, 10.1073/pnas.0909142106. PMC2757821 19805370

[19] I. Bjorkhem , “Crossing the Barrier: Oxysterols as Cholesterol Transporters and Metabolic Modulators in the Brain,” Journal of Internal Medicine 260, no. 6 (2006): 493–508, 10.1111/j.1365-2796.2006.01725.x. 17116000

[20] V. Mutemberezi , O. Guillemot‐Legris , and G. G. Muccioli , “Oxysterols: from Cholesterol Metabolites to Key Mediators,” Progress in Lipid Research 64 (2016): 152–169, 10.1016/j.plipres.2016.09.002. 27687912

[21] M. K. Cowman , H. G. Lee , K. L. Schwertfeger , J. B. McCarthy , and E. A. Turley , “The Content and Size of Hyaluronan in Biological Fluids and Tissues,” Frontiers in Immunology 6 (2015): 261, 10.3389/fimmu.2015.00261. 26082778 PMC4451640

[22] D. Jiang , J. Liang , and P. W. Noble , “Hyaluronan in Tissue Injury and Repair,” Annual Review of Cell and Developmental Biology 23 (2007): 435–461, 10.1146/annurev.cellbio.23.090506.123337. 17506690

[23] L. A. Warren and D. J. Rossi , “Stem Cells and Aging in the Hematopoietic System,” Mechanisms of Ageing and Development 130, no. 1‐2 (2009): 46–53, 10.1016/j.mad.2008.03.010. 18479735 PMC3992834

[24] D. J. Rossi , D. Bryder , J. M. Zahn , et al., “Cell Intrinsic Alterations Underlie Hematopoietic Stem Cell Aging,” Proceedings of the National Academy of Sciences 102, no. 26 (2005): 9194–9199, 10.1073/pnas.0503280102. PMC1153718 15967997

[25] S. Caballero , D. L. Kent , N. Sengupta , et al., “Bone Marrow–Derived Cell Recruitment to the Neurosensory Retina and Retinal Pigment Epithelial Cell Layer Following Subthreshold Retinal Phototherapy,” Investigative Opthalmology & Visual Science 58, no. 12 (2017): 5164–5176, 10.1167/iovs.16-20736. PMC5636205 29049716

[26] J. A. Mazzitelli , F. E. Pulous , L. C. D. Smyth , et al., “Skull Bone Marrow Channels as Immune Gateways to the central Nervous System,” Nature Neuroscience 26, no. 12 (2023): 2052–2062. 37996526 10.1038/s41593-023-01487-1 PMC10894464

[27] T. Croese , G. Castellani , and M. Schwartz , “Immune Cell Compartmentalization for Brain Surveillance and Protection,” Nature Immunology 22, no. 9 (2021): 1083–1092. 34429552 10.1038/s41590-021-00994-2

[28] Z. I. Kolabas , L. B. Kuemmerle , R. Perneczky , et al., “Distinct Molecular Profiles of Skull Bone Marrow in Health and Neurological Disorders,” Cell 186, no. 17 (2023): 3706–3725. 37562402 10.1016/j.cell.2023.07.009 PMC10443631

[29] S. Pinho and P. S. Frenette , “Haematopoietic Stem Cell Activity and Interactions with the Niche,” Nature Reviews Molecular Cell Biology 20, no. 5 (2019): 303–320, 10.1038/s41580-019-0103-9. 30745579 PMC6483843

[30] P. Wostyn , V. De Groot , D. Van Dam , K. Audenaert , H. E. Killer , and P. P. De Deyn , “The Glymphatic Hypothesis of Glaucoma: a Unifying Concept Incorporating Vascular, Biomechanical, and Biochemical Aspects of the Disease,” BioMed Research International 2017 (2017): 5123148, 10.1155/2017/5123148. 28948167 PMC5602488

[31] B. Asare‐Bediako , Y. Adu‐Agyeiwaah , A. Abad , et al., “Hematopoietic Cells Influence Vascular Development in the Retina,” Cells 11, no. 20 (2022): 3207. 36291075 10.3390/cells11203207 PMC9601270

[32] S. Botolin and L. R. McCabe , “Bone Loss and Increased Bone Adiposity in Spontaneous and Pharmacologically Induced Diabetic Mice,” Endocrinology 148, no. 1 (2007): 198–205, 10.1210/en.2006-1006. 17053023

[33] E. Keats and Z. A. Khan , “Unique Responses of Stem Cell‐derived Vascular Endothelial and Mesenchymal Cells to High Levels of Glucose,” PLoS ONE 7, no. 6 (2012): 38752, 10.1371/journal.pone.0038752. PMC3368917 22701703

[34] J. L. Johns and M. M. Christopher , “Extramedullary Hematopoiesis: a New Look at the Underlying Stem Cell Niche, Theories of Development, and Occurrence in Animals,” Veterinary Pathology 49, no. 3 (2012): 508–523, 10.1177/0300985811432344. 22262354

[35] C. P. Vieira , S. D. Fortmann , M. Hossain , et al., “Selective LXR Agonist DMHCA Corrects Retinal and Bone Marrow Dysfunction in Type 2 Diabetes,” JCI insight 5, no. 13 (2020): 137230. 32641586 10.1172/jci.insight.137230 PMC7406260

[36] Y. Duan , E. Beli , S. Li Calzi , et al., “Loss of Angiotensin‐Converting Enzyme 2 Exacerbates Diabetic Retinopathy by Promoting Bone Marrow Dysfunction,” Stem Cells 36, no. 9 (2018): 1430–1440, 10.1002/stem.2848. 29761600 PMC6410700

[37] A. D. Bhatwadekar , Y. Duan , M. Korah , et al., “Hematopoietic Stem/Progenitor Involvement in Retinal Microvascular Repair during Diabetes: Implications for Bone Marrow Rejuvenation,” Vision Research 139 (2017): 211–220, 10.1016/j.visres.2017.06.016. 29042190 PMC6335030

[38] H. Chakravarthy , E. Beli , S. Navitskaya , et al., “Imbalances in Mobilization and Activation of Pro‐Inflammatory and Vascular Reparative Bone Marrow‐Derived Cells in Diabetic Retinopathy,” PLoS ONE 11, no. 1 (2016): 0146829, 10.1371/journal.pone.0146829. PMC4711951 26760976

[39] J. M. Dominguez , M. A. Yorek , and M. B. Grant , “Combination Therapies Prevent the Neuropathic, Proinflammatory Characteristics of Bone Marrow in Streptozotocin‐induced Diabetic Rats,” Diabetes 64, no. 2 (2015): 643–653, 10.2337/db14-0433. 25204979 PMC4876792

[40] P. Hu , J. S. Thinschmidt , Y. Yan , et al., “CNS Inflammation and Bone Marrow Neuropathy in Type 1 Diabetes,” The American Journal of Pathology 183, no. 5 (2013): 1608–1620, 10.1016/j.ajpath.2013.07.009. 24160325 PMC3814523

[41] S. Hazra , Y. P. Jarajapu , V. Stepps , et al., “Long‐term Type 1 Diabetes Influences Haematopoietic Stem Cells by Reducing Vascular Repair Potential and Increasing Inflammatory Monocyte Generation in a Murine Model,” Diabetologia 56, no. 3 (2013): 644–653, 10.1007/s00125-012-2781-0. 23192694 PMC3773610

[42] F. Ferraro , S. Lymperi , S. Mendez‐Ferrer , et al., “Diabetes Impairs Hematopoietic Stem Cell Mobilization by Altering Niche Function,” Science Translational Medicine 3, no. 104 (2011), 10.1126/scitranslmed.3002191. PMC3754876 21998408

[43] S. Hazra , A. Rasheed , A. Bhatwadekar , et al., “Liver X Receptor Modulates Diabetic Retinopathy Outcome in a Mouse Model of Streptozotocin‐induced Diabetes,” Diabetes 61, no. 12 (2012): 3270–3279, 10.2337/db11-1596. 22891211 PMC3501845

[44] M. Hanoun , M. Maryanovich , A. Arnal‐Estape , and P. S. Frenette , “Neural Regulation of Hematopoiesis, Inflammation, and Cancer,” Neuron 86, no. 2 (2015): 360–373, 10.1016/j.neuron.2015.01.026. 25905810 PMC4416657

[45] X. Zhao , M. Yao , Y. Wang , et al., “Neuroregulation during Bone Formation and Regeneration: Mechanisms and Strategies,” ACS Appl Mater Interfaces 17, no. 5 (2025): 7223–7250. 39869030 10.1021/acsami.4c16786

[46] L. Chen , Y. Yang , J. Chen , et al., “Intranasal Delivery of Hypoxia‐preconditioned Extracellular Vesicles Derived from BMSCs Alleviates Neuroinflammation and Brain Dysfunction in TBI,” Stem Cell Res Ther 16, no. 1 (2025): 1–33. 41057947 10.1186/s13287-025-04572-3 PMC12506410

[47] A. D. Bhatwadekar , E. P. Guerin , Y. P. Jarajapu , et al., “Transient Inhibition of Transforming Growth Factor‐β1 in Human Diabetic CD34+ Cells Enhances Vascular Reparative Functions,” Diabetes 59, no. 8 (2010): 2010–2019, 10.2337/db10-0287. 20460428 PMC2911069

[48] S. Rathinavelu , C. Guidry‐Elizondo , and J. Banu , “Molecular Modulation of Osteoblasts and Osteoclasts in Type 2 Diabetes,” Journal of Diabetes Research 2018 (2018): 6354787, 10.1155/2018/6354787. 30525054 PMC6247387

[49] K. Lin , L. Yang , Y. Xiong , K. Feng , W. Zeng , and B. Deng , “Plasma C1q/Tumor Necrosis Factor‐related Protein‐3 Concentrations Are Associated with Diabetic Peripheral Neuropathy,” BMJ Open Diabetes Res Care 10, no. 2 (2022): 002746. 10.1136/bmjdrc-2021-002746 PMC8984060 35383102

[50] A. V. Schwartz and D. E. Sellmeyer , “Women, Type 2 Diabetes, and Fracture Risk,” Current Diabetes Reports 4, no. 5 (2004): 364–369, 10.1007/s11892-004-0039-z. 15461902

[51] G. Spinetti , D. Cordella , O. Fortunato , et al., “Global Remodeling of the Vascular Stem Cell Niche in Bone Marrow of Diabetic Patients,” Circulation Research 112, no. 3 (2013): 510–522, 10.1161/CIRCRESAHA.112.300598. 23250986 PMC3616365

[52] G. P. Fadini , M. Rigato , R. Cappellari , B. M. Bonora , and A. Avogaro , “Long‐term Prediction of Cardiovascular Outcomes by Circulating CD34+ and CD34+CD133+ Stem Cells in Patients with Type 2 Diabetes,” Diabetes Care 40, no. 1 (2017): 125–131, 10.2337/dc16-1755. 27815289

[53] A. M. Plummer , A. T. Culbertson , and M. Liao , “The ABCs of Sterol Transport,” Annual Review of Physiology 83 (2021): 153–181, 10.1146/annurev-physiol-031620-094944. 33141631

[54] M. B. Grant , W. S. May , S. Caballero , et al., “Adult Hematopoietic Stem Cells Provide Functional Hemangioblast Activity during Retinal Neovascularization,” Nature Medicine 8, no. 6 (2002): 607–612, 10.1038/nm0602-607. 12042812

[55] T. Kurimoto , Y. Yin , G. Habboub , et al., “Neutrophils Express Oncomodulin and Promote Optic Nerve Regeneration,” The Journal of Neuroscience 33, no. 37 (2013): 14816–14824, 10.1523/JNEUROSCI.5511-12.2013. 24027282 PMC3771038

[56] P. Wostyn , D. Van Dam , K. Audenaert , H. E. Killer , P. P. De Deyn , and V. De Groot , “A New Glaucoma Hypothesis: A Role of Glymphatic System Dysfunction,” Fluids Barriers CNS 12, no. 1 (2015): 16. 26118970 10.1186/s12987-015-0012-z PMC4485867

[57] E. Dzierzak and A. Bigas , “Blood Development: Hematopoietic Stem Cell Dependence and Independence,” Cell Stem Cell 22, no. 5 (2018): 639–651, 10.1016/j.stem.2018.04.015. 29727679

[58] L. G. Schuettpelz and D. C. Link , “Regulation of Hematopoietic Stem Cell Activity by Inflammation,” Frontiers in Immunology 4 (2013): 204, 10.3389/fimmu.2013.00204. 23882270 PMC3715736

[59] P. J. D. Delhanty and J. A. Visser , “Navigating the Strengths and Constraints of Mouse Models in Obesity Research,” Endocrinology 166, no. 9 (2025): bqaf123. 40702736 10.1210/endocr/bqaf123 PMC12342185

[60] B. Asare‐Bediako , S. K. Noothi , S. Li Calzi , et al., “Characterizing the Retinal Phenotype in the High‐Fat Diet and Western Diet Mouse Models of Prediabetes,” Cells 9, no. 2 (2020): 464. 32085589 10.3390/cells9020464 PMC7072836

[61] M. Blaszkiewicz , K. M‐A , J. Peng , J. McGregor , and K. L. Townsend , “Obesity‐Induced Idiopathic Intracranial Hypertension (IIH) Is Associated With a Distinct Pattern of Metabolite Expression in Cerebrospinal Fluid (CSF) Indicative of Future Risk for Neurodegeneration,” iScience (2025), 10.2139/ssrn.5495745.

[62] R. Prasad , J. L. Floyd , M. Dupont , et al., “Maintenance of Enteral ACE2 Prevents Diabetic Retinopathy in Type 1 Diabetes,” Circulation Research 132, no. 1 (2023): e1–e21. 36448480 10.1161/CIRCRESAHA.122.322003 PMC9822874

[63] A. Limaye , “Drishti: a Volume Exploration and Presentation Tool,” in Developments in X‐ray Tomography VIII, (SPIE, 2012).

[64] Y. Hu , A. Limaye , and J. Lu , “Three‐dimensional Segmentation of Computed Tomography Data Using Drishti Paint: New Tools and Developments,” Royal Society Open Science 7, no. 12 (2020): 201033. 33489265 10.1098/rsos.201033 PMC7813226

[65] R. Domander , A. A. Felder , and M. Doube , “BoneJ2 ‐ refactoring Established Research Software,” Wellcome Open Research 6 (2021): 37, 10.12688/wellcomeopenres.16619.1. 33954267 PMC8063517

[66] J. Schindelin , I. Arganda‐Carreras , E. Frise , et al., “an Open‐source Platform for Biological‐image Analysis,” Nature Methods 9, no. 7 (2012): 676–682. 22743772 10.1038/nmeth.2019 PMC3855844

[67] S. S. Park , S. Caballero , G. Bauer , et al., “Long‐Term Effects of Intravitreal Injection of GMP‐Grade Bone‐Marrow–Derived CD34+ Cells in NOD‐SCID Mice with Acute Ischemia‐Reperfusion Injury,” Investigative Opthalmology & Visual Science 53, no. 2 (2012): 986–994, 10.1167/iovs.11-8833. PMC3317435 22247454

[68] S. Caballero , N. Sengupta , A. Afzal , et al., “Ischemic Vascular Damage Can be Repaired by Healthy, but Not Diabetic, Endothelial Progenitor Cells,” Diabetes 56, no. 4 (2007): 960–967, 10.2337/db06-1254. 17395742 PMC3746188

[69] S. R. Chartier , S. A. Mitchell , L. A. Majuta , and P. W. Mantyh , “The Changing Sensory and Sympathetic Innervation of the Young, Adult and Aging Mouse Femur,” Neuroscience 387 (2018): 178–190, 10.1016/j.neuroscience.2018.01.047. 29432884 PMC6086773

[70] S. L. Calzi , D. Chakraborty , P. Hu , et al., “Targeting Diabetic Retinopathy with Human iPSC‐Derived Vascular Reparative Cells in a Type 2 Diabetes Model,” Cells 14, no. 17 (2025): 1352. 40940763 10.3390/cells14171352 PMC12428381

[71] V. Matyash , G. Liebisch , T. V. Kurzchalia , A. Shevchenko , and D. Schwudke , “Lipid Extraction by Methyl‐tert‐butyl Ether for High‐throughput Lipidomics,” Journal of Lipid Research 49, no. 5 (2008): 1137–1146, 10.1194/jlr.D700041-JLR200. 18281723 PMC2311442

[72] R. E. Patterson , A. J. Ducrocq , D. J. McDougall , T. J. Garrett , and R. A. Yost , “Comparison of Blood Plasma Sample Preparation Methods for Combined LC–MS Lipidomics and Metabolomics,” Journal of Chromatography B 1002 (2015): 260–266, 10.1016/j.jchromb.2015.08.018. PMC4582001 26343017

