# Supplementary material for: Resilient Calvarial Bone Marrow Supports Retinal Repair in Type 2 Diabetes
Source: Adv Sci (Weinh). 2026 Jan 4;13(13):e19680. doi: 10.1002/advs.202519680 (PMC12955880; doi:10.1002/advs.202519680)
Supplement: Supplementary file 1 — Supporting File 1: advs73436‐sup‐0001‐Figures.pptx. [file ADVS-13-e19680-s003.pptx]

## Slide 1
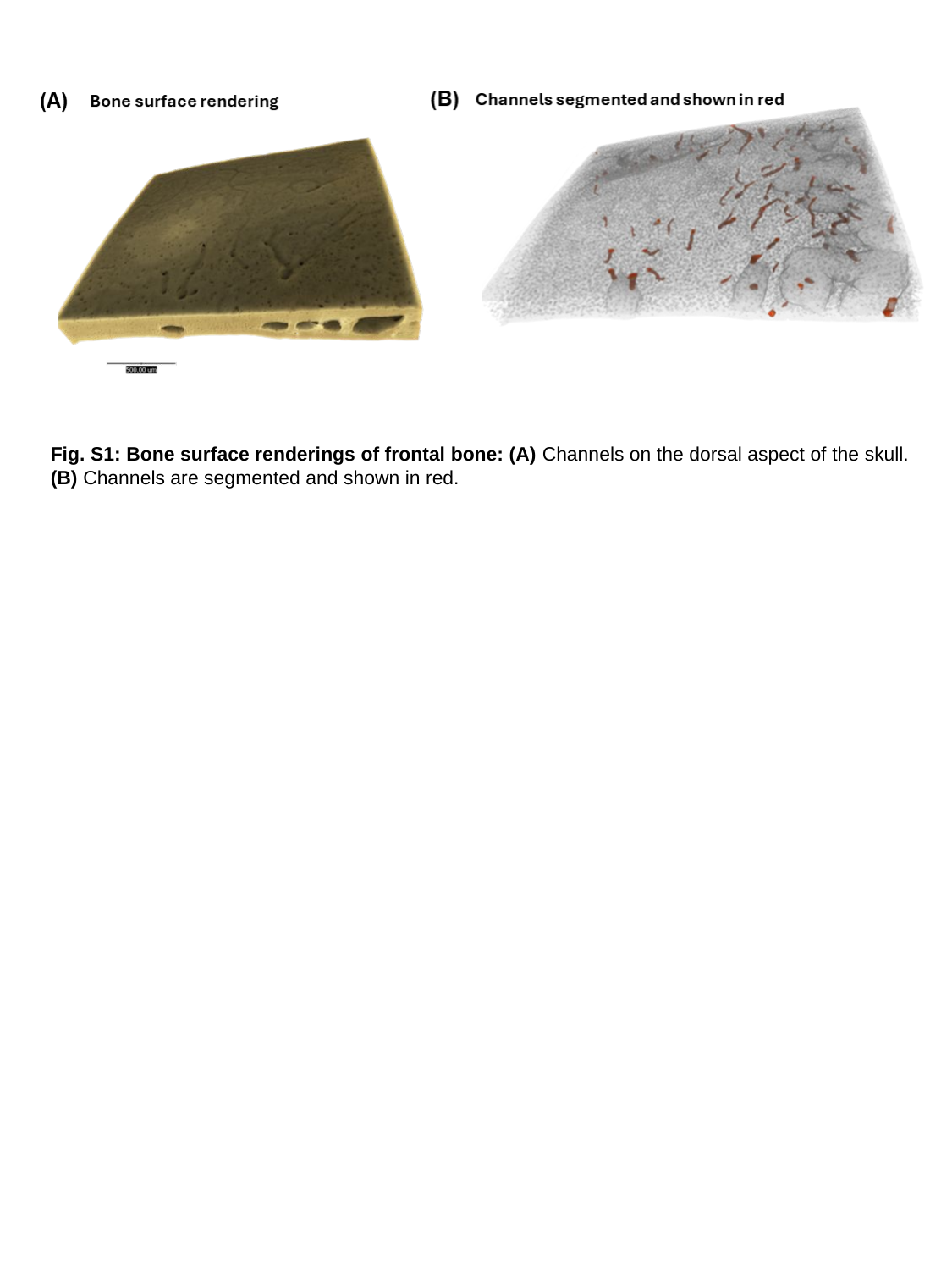

Fig. S1: Bone surface renderings of frontal bone: (A) Channels on the dorsal aspect of the skull. (B) Channels are segmented and shown in red.

## Slide 2
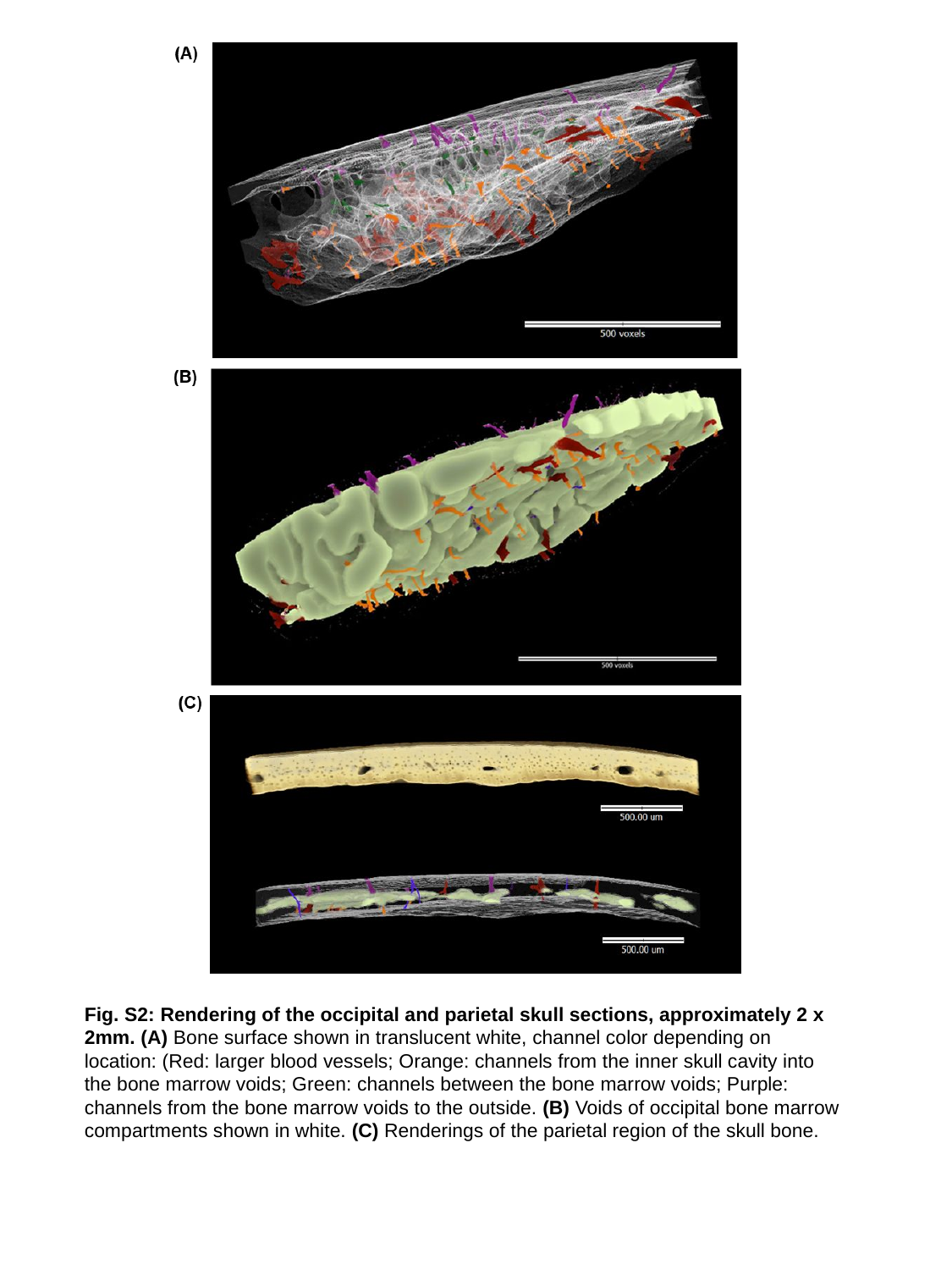

Fig. S2: Rendering of the occipital and parietal skull sections, approximately 2 x 2mm. (A) Bone surface shown in translucent white, channel color depending on location: (Red: larger blood vessels; Orange: channels from the inner skull cavity into the bone marrow voids; Green: channels between the bone marrow voids; Purple: channels from the bone marrow voids to the outside. (B) Voids of occipital bone marrow compartments shown in white. (C) Renderings of the parietal region of the skull bone.

## Slide 3
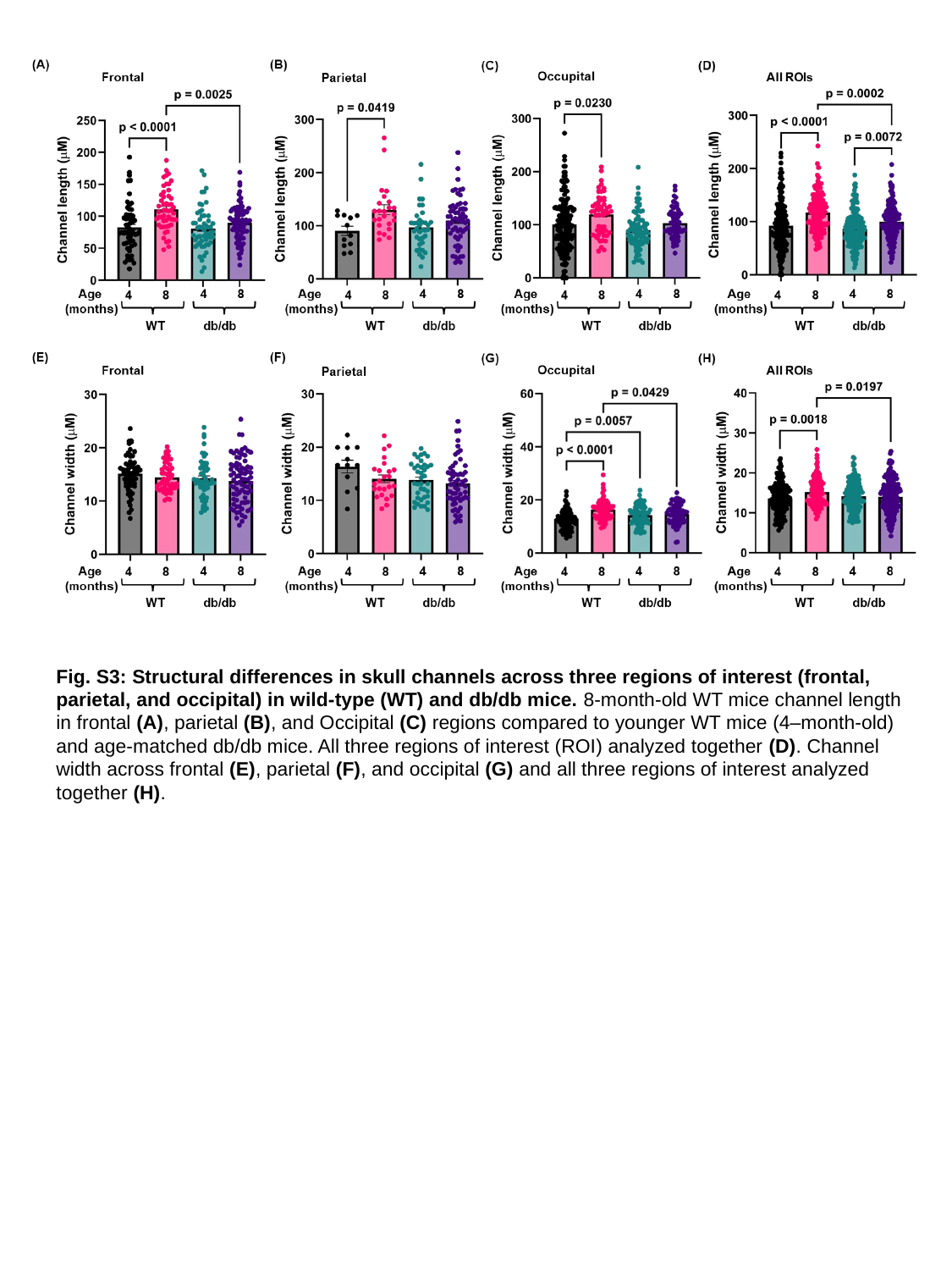

Fig. S3: Structural differences in skull channels across three regions of interest (frontal, parietal, and occipital) in wild-type (WT) and db/db mice. 8-month-old WT mice channel length in frontal (A), parietal (B), and Occipital (C) regions compared to younger WT mice (4–month-old) and age-matched db/db mice. All three regions of interest (ROI) analyzed together (D). Channel width across frontal (E), parietal (F), and occipital (G) and all three regions of interest analyzed together (H).

## Slide 4
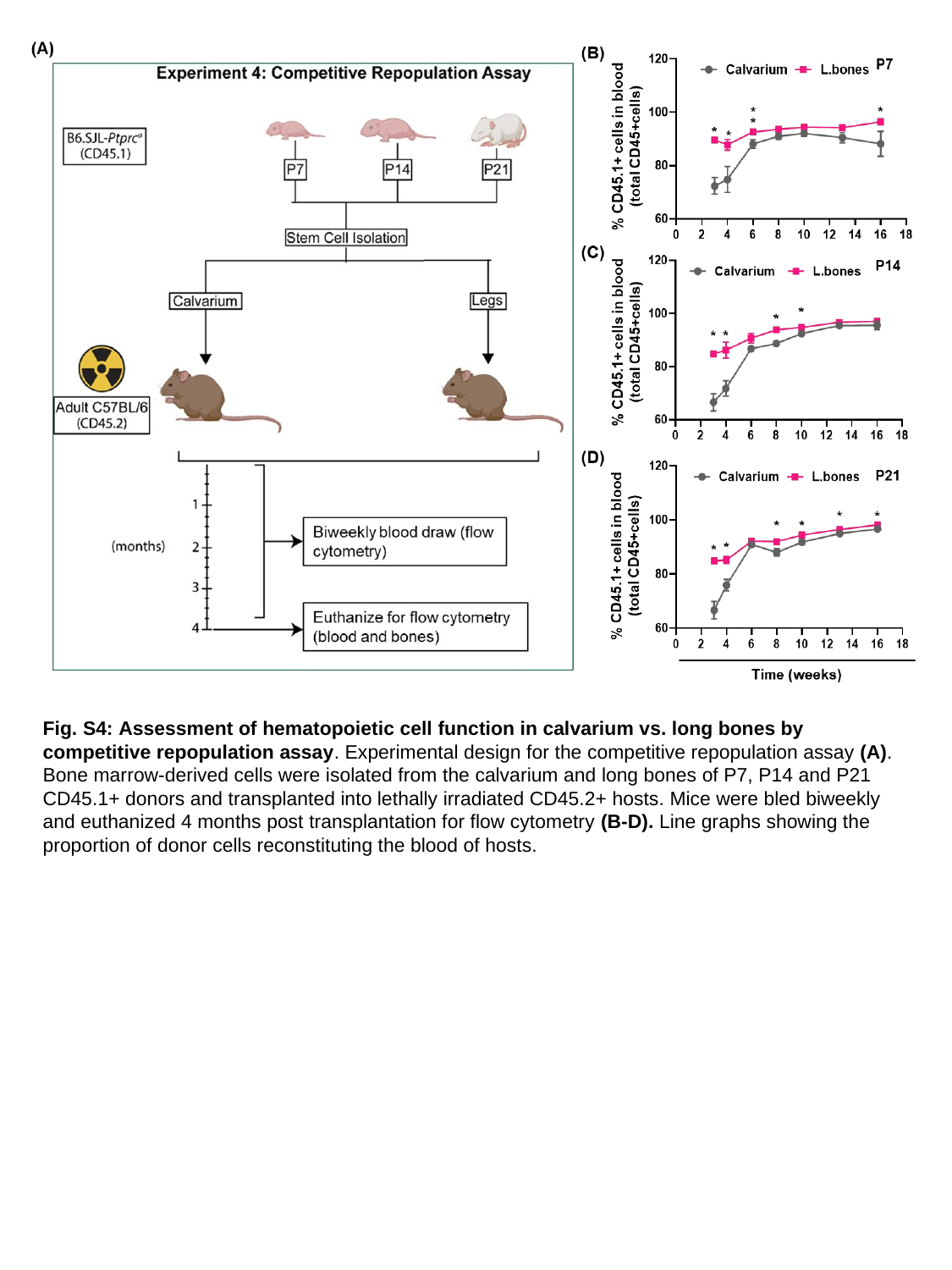

Fig. S4: Assessment of hematopoietic cell function in calvarium vs. long bones by competitive repopulation assay. Experimental design for the competitive repopulation assay (A). Bone marrow-derived cells were isolated from the calvarium and long bones of P7, P14 and P21 CD45.1+ donors and transplanted into lethally irradiated CD45.2+ hosts. Mice were bled biweekly and euthanized 4 months post transplantation for flow cytometry (B-D). Line graphs showing the proportion of donor cells reconstituting the blood of hosts.

## Slide 5
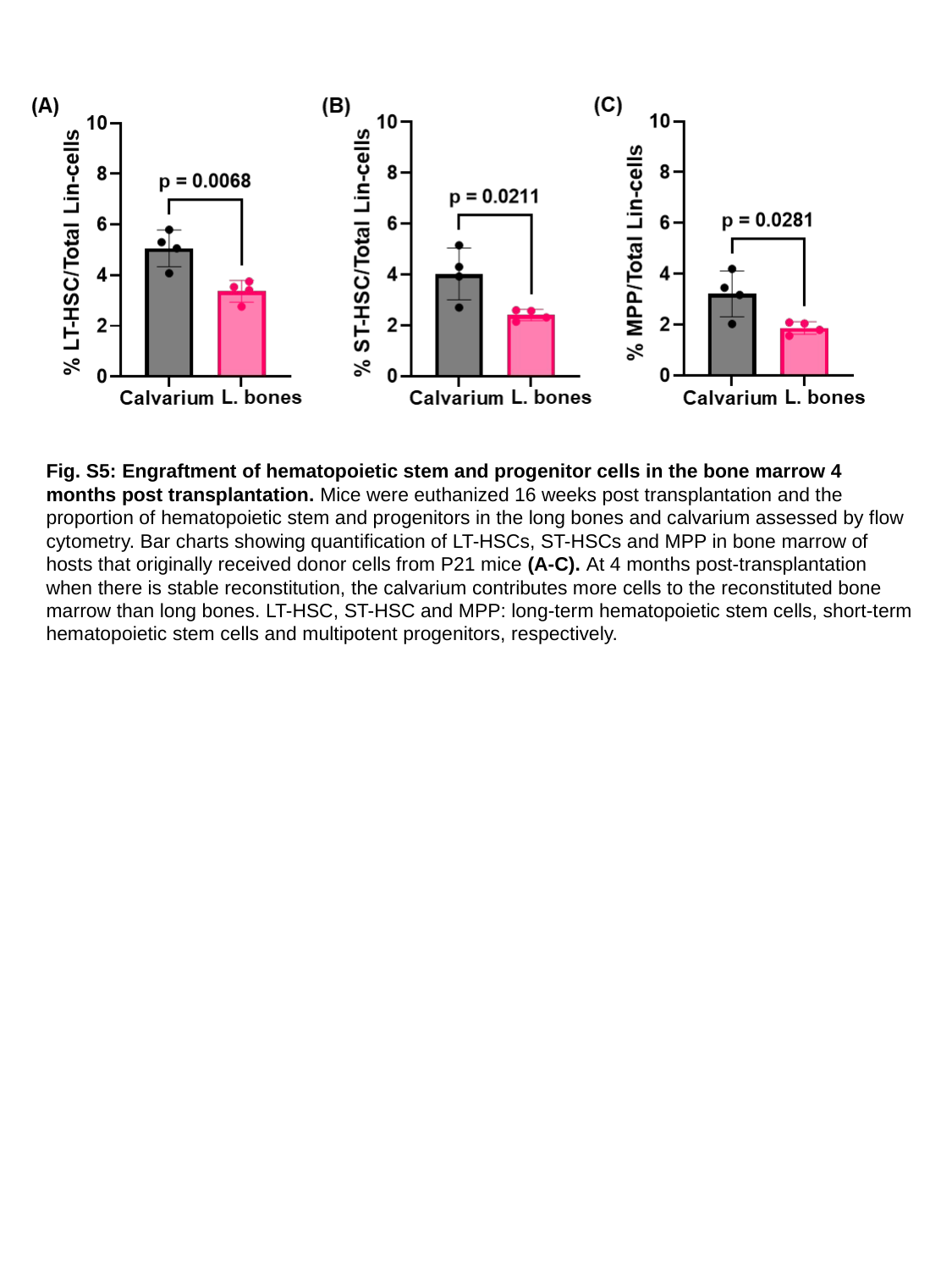

Fig. S5: Engraftment of hematopoietic stem and progenitor cells in the bone marrow 4 months post transplantation. Mice were euthanized 16 weeks post transplantation and the proportion of hematopoietic stem and progenitors in the long bones and calvarium assessed by flow cytometry. Bar charts showing quantification of LT-HSCs, ST-HSCs and MPP in bone marrow of hosts that originally received donor cells from P21 mice (A-C). At 4 months post-transplantation when there is stable reconstitution, the calvarium contributes more cells to the reconstituted bone marrow than long bones. LT-HSC, ST-HSC and MPP: long-term hematopoietic stem cells, short-term hematopoietic stem cells and multipotent progenitors, respectively.

## Slide 6
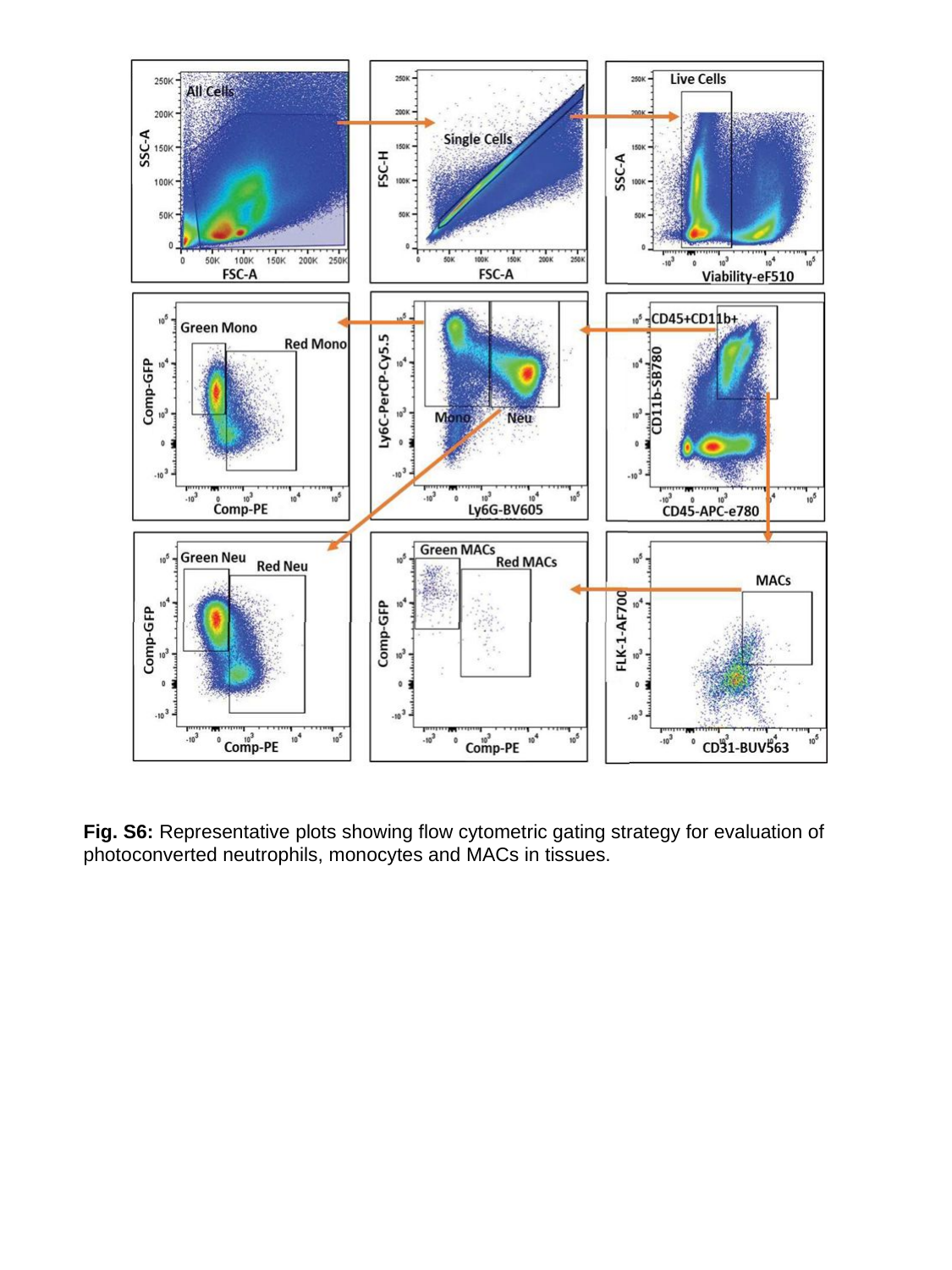

Fig. S6: Representative plots showing flow cytometric gating strategy for evaluation of photoconverted neutrophils, monocytes and MACs in tissues.
76

## Slide 7
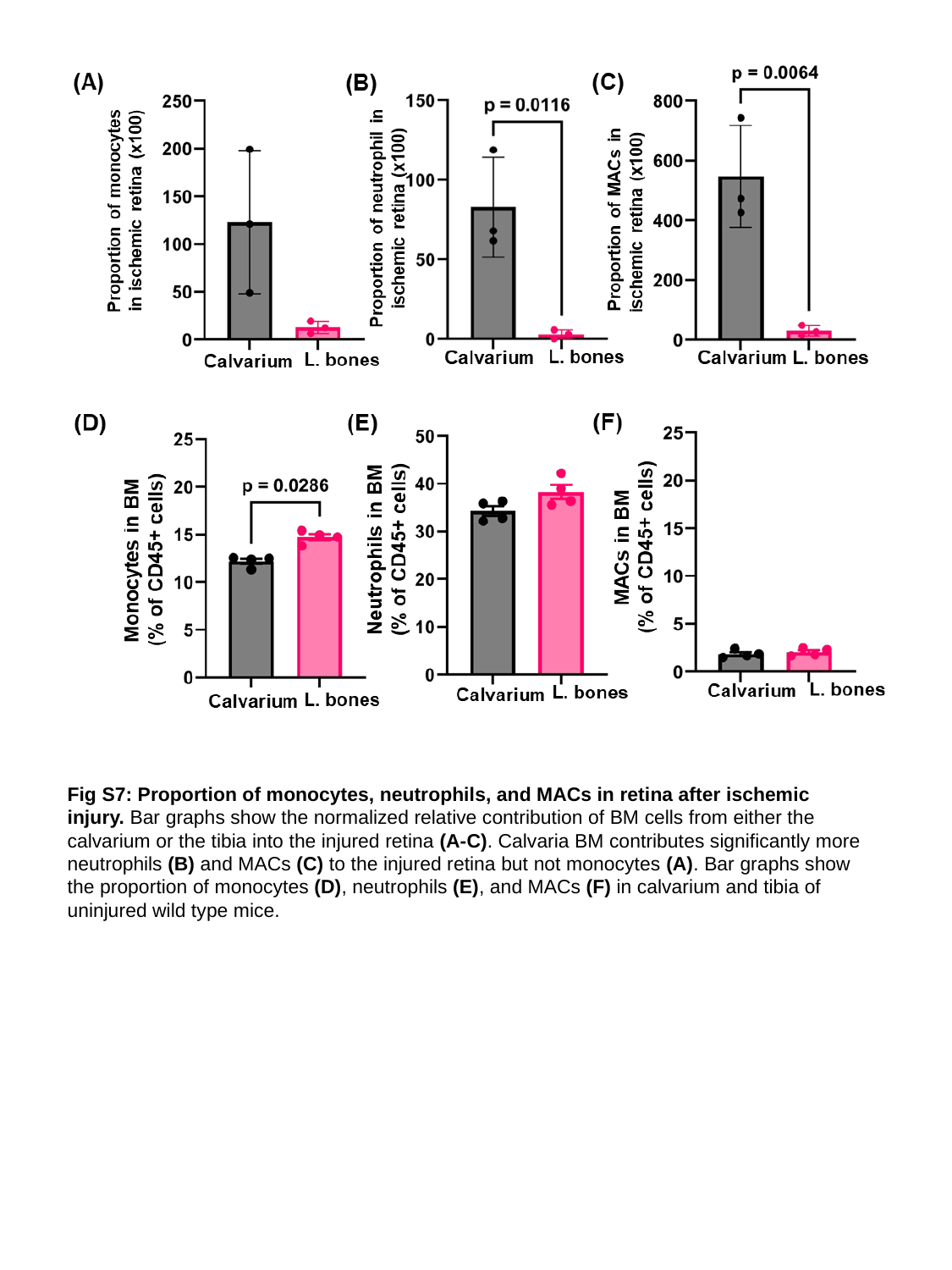

Fig S7: Proportion of monocytes, neutrophils, and MACs in retina after ischemic injury. Bar graphs show the normalized relative contribution of BM cells from either the calvarium or the tibia into the injured retina (A-C). Calvaria BM contributes significantly more neutrophils (B) and MACs (C) to the injured retina but not monocytes (A). Bar graphs show the proportion of monocytes (D), neutrophils (E), and MACs (F) in calvarium and tibia of uninjured wild type mice.
