# Supplementary material for: Resilient Calvarial Bone Marrow Supports Retinal Repair in Type 2 Diabetes
Source: Adv Sci (Weinh). 2026 Jan 4;13(13):e19680. doi: 10.1002/advs.202519680 (PMC12955880; doi:10.1002/advs.202519680)
Supplement: Supplementary file 2 — Supporting File 2: advs73436‐sup‐0002‐Table S1.docx. [file ADVS-13-e19680-s001.docx]

**Supplementary Table S1: A list of flow cytometry antibodies**

| Target cell population | Markers | Clone | Source | Catalog # | Working dilution (μL) |
| --- | --- | --- | --- | --- | --- |
| Stem Cell panel | CD45 BUV615-P | 30-F11 | BD Biosciences USA | 751170 | 1:100 |
|  | c-Kit (CD117) eFluor 450 | 2B8 | Invitrogen USA | 48-1171-82 | 1:100 |
|  | FcγRII/III (CD16/32) BV605 | 93 | Invitrogen USA | 63-0161-82 | 1:100 |
|  | Sca-1 BV650 | D7 | Invitrogen USA | 64-5981-82 | 1:100 |
|  | Hematopoietic Lineage (Lin) (CD3, CD45R, CD11b, TER-119, Gr-1) FITC |  | Invitrogen USA | 22-7770-72 | 1:100 |
|  | Flt3 BB700 | BV10A4H2 | Invitrogen USA | 17-1357-41 | 1:100 |
|  | CD34 PE | MEC14.7 | Biolegend USA | 119308 | 1:100 |
|  | CD127 APC-eFluor 780 | A7E34 | Invitrogen USA | 47-1271-82 | 1:100 |
|  | Live/Dead | Sytox Blue | Invitrogen USA | S34857 | 1:100 |
| Myeloid panel | CD45 BUV615-P | 30-F11 | BD Biosciences USA | 751170 | 1:100 |
|  | Ly6G BV 605 | 1A8 | Biolegend USA | 127639 | 1:100 |
|  | Ly6C APC-Cy7 | HK1.4 | Invitrogen USA | 47-5932-82 | 1:100 |
|  | CD11b BV780 | M1/70 | Invitrogen USA | 78-0112-82 | 1:100 |
|  | CCR2 FITC | SA203G11 | BD Biosciences USA | 150608 | 1:100 |
|  | Flk-1 PercP Cy5.5 | Avas12a1 | BD Biosciences USA | 560681 | 1:100 |
|  | CD31 PE | 390 | Invitrogen USA | 12-0311-82 | 1:100 |
|  | CD206 APC | MR6F3 | Invitrogen USA | 17-2061-82 | 1:100 |
|  | F4/80 eFluor 450 | BM8 | Invitrogen USA | 48-4801-82 | 1:100 |
|  | Live/Dead | Sytox Blue | Invitrogen USA | S34857 | 1:100 |
