# Supplementary figures and images for: Resilient Calvarial Bone Marrow Supports Retinal Repair in Type 2 Diabetes
Source: Adv Sci (Weinh). 2026 Jan 4;13(13):e19680. doi: 10.1002/advs.202519680 (PMC12955880; doi:10.1002/advs.202519680)

## Slide 1
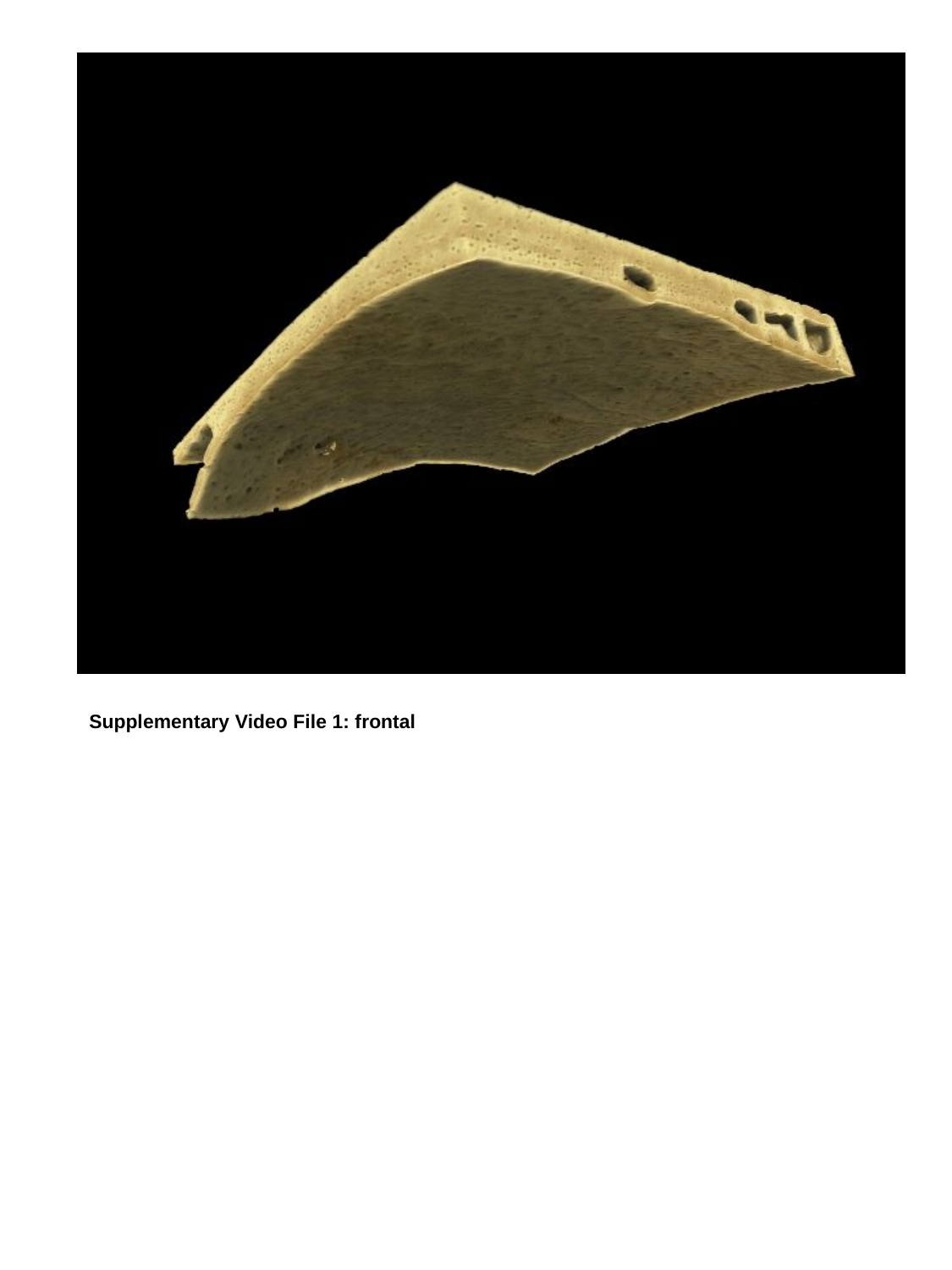

Supplementary Video File 1: frontal

Supplement: Supplementary file 3 — Supporting File 3: advs73436‐sup‐0003‐Video S1.pptx. [file ADVS-13-e19680-s004.pptx]

## Slide 1
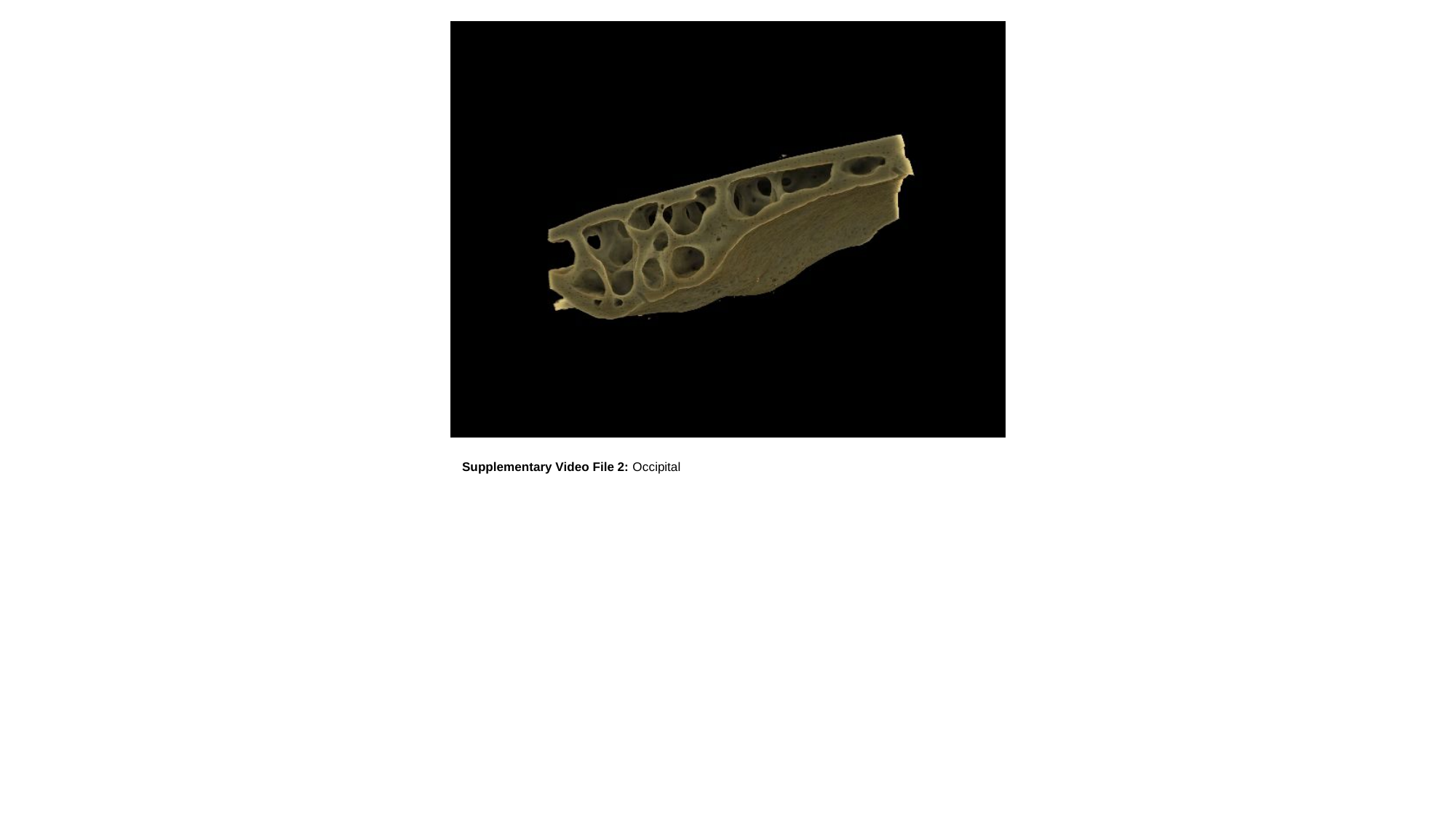

Supplementary Video File 2: Occipital

Supplement: Supplementary file 4 — Supporting File 4: advs73436‐sup‐0004‐Video S2.pptx. [file ADVS-13-e19680-s002.pptx]

## Slide 1
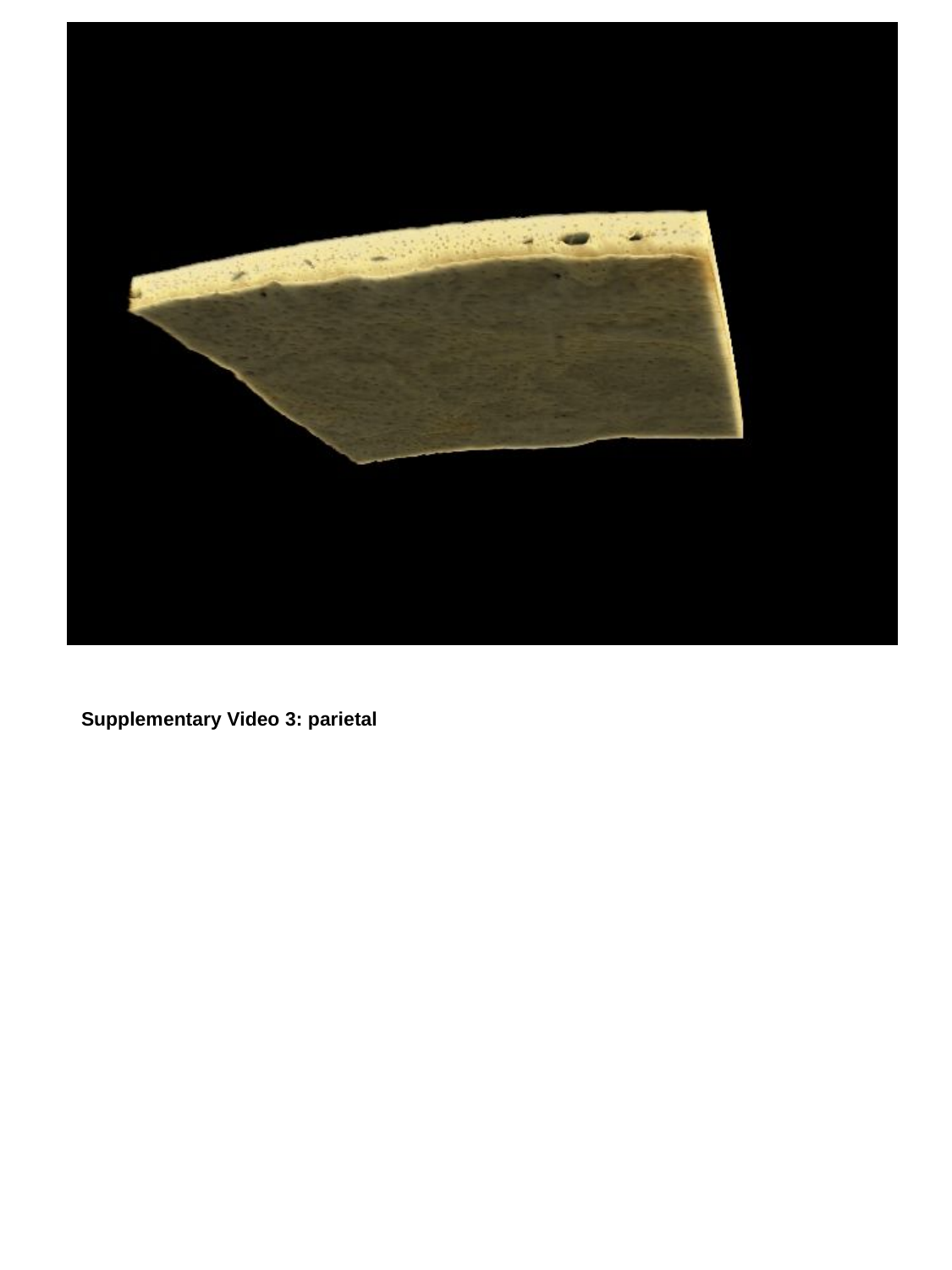

Supplementary Video 3: parietal

Supplement: Supplementary file 5 — Supporting File 5: advs73436‐sup‐0005‐Video S3.pptx. [file ADVS-13-e19680-s005.pptx]
